# In Vitro, In Vivo and In Silico Characterization of a Novel Kappa-Opioid Receptor Antagonist

**DOI:** 10.3390/ph15060680

**Published:** 2022-05-28

**Authors:** Kristina Puls, Aina-Leonor Olivé-Marti, Szymon Pach, Birgit Pinter, Filippo Erli, Gerhard Wolber, Mariana Spetea

**Affiliations:** 1 Department of Pharmaceutical Chemistry, Institute of Pharmacy, Freie Universität Berlin, Königin-Luise-Str. 2-4, 14195 Berlin, Germany; kristina.puls@fu-berlin.de (K.P.); s.pach@fu-berlin.de (S.P.); 2 Department of Pharmaceutical Chemistry, Institute of Pharmacy and Center for Molecular Biosciences Innsbruck (CMBI), University of Innsbruck, Innrain 80-82, 6020 Innsbruck, Austria; aina-leonor.olive-marti@uibk.ac.at (A.-L.O.-M.); birgit-pinter@gmax.at (B.P.); f.erli@hotmail.it (F.E.)

**Keywords:** GPCRs, kappa-opioid receptor, antagonists, binding affinity, selectivity, in vivo antagonism, molecular docking, molecular dynamics simulations, dynophores

## Abstract

Kappa-opioid receptor (KOR) antagonists are promising innovative therapeutics for the treatment of the central nervous system (CNS) disorders. The new scaffold opioid ligand, Compound A, was originally found as a mu-opioid receptor (MOR) antagonist but its binding/selectivity and activation profile at the KOR and delta-opioid receptor (DOR) remain elusive. In this study, we present an in vitro, in vivo and in silico characterization of Compound A by revealing this ligand as a KOR antagonist in vitro and in vivo. In the radioligand competitive binding assay, Compound A bound at the human KOR, albeit with moderate affinity, but with increased affinity than to the human MOR and without specific binding at the human DOR, thus displaying a preferential KOR selectivity profile. Following subcutaneous administration in mice, Compound A effectively reverse the antinociceptive effects of the prototypical KOR agonist, U50,488. In silico investigations were carried out to assess the structural determinants responsible for opioid receptor subtype selectivity of Compound A. Molecular docking, molecular dynamics simulations and dynamic pharmacophore (dynophore) generation revealed differences in the stabilization of the chlorophenyl moiety of Compound A within the opioid receptor binding pockets, rationalizing the experimentally determined binding affinity values. This new chemotype bears the potential for favorable ADMET properties and holds promise for chemical optimization toward the development of potential therapeutics.

## 1. Introduction

Opioid receptors belong to the large family of G protein-coupled receptors (GPCRs) [1]. GPCRs are membrane-embedded receptors that share a seven-transmembrane (7TM) helical structure and elicit a myriad of biological activities upon activation by endogenous or exogenous ligands [2,3,4]. Thus, GPCRs are widely addressed targets for drug development with around one-third of all approved drugs targeting GPCRs [5]. To date, the human opioid receptor family consists of four receptor subtypes, namely, the kappa-, mu- and delta-opioid receptors (KOR, MOR and DOR, respectively), and the non-classical nociceptin/orphanin FQ peptide (NOP) receptor [1,6]. Opioid receptors have a distinct expression pattern throughout the central and peripheral nervous systems (CNS and PNS) and are involved in the regulation of pain, response to stress, reward processing and regulation of mood states, among many other functions [1,6,7,8,9].

**Figure 1:**
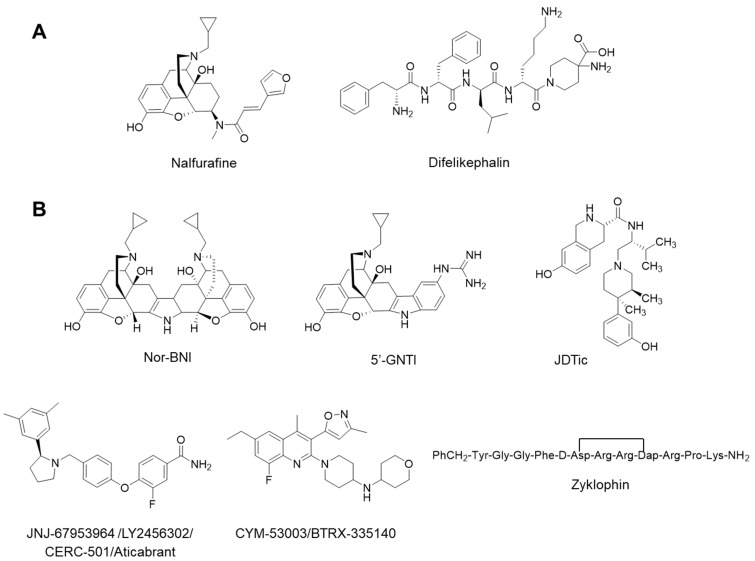
Representative selective KOR ligands used in the clinics, as potential therapeutics or research tools. (**A**) KOR agonists; (**B**, **top**) long-acting KOR antagonists; (**B**, **bottom**) short-acting KOR antagonists.

Over many years, the MOR has been the main pharmacological target for effective pain relief and treatment of other pathophysiological conditions, such as drug addiction and gastrointestinal motility disorders [1,10,11,12]. However, the MOR is also the target of the most misused and abused opioid drugs, resulting in an ongoing and rapidly emerging opioid epidemic worldwide [13,14]. Therefore, the KOR has recently gained increased attention as a prominent GPCR in the pursuit of novel pharmacotherapies for a variety of human diseases, due to its role in mediating many physiological and pathophysiological responses [15]. Activation of the KOR is viewed as a promising strategy for the treatment of pain, itch and epilepsy, whereas receptor blockade is associated with potential therapeutic effects in mood (depression and anxiety) and addictive disorders [16,17,18,19,20,21]. Selective ligands for the KOR with diverse scaffolds—such as small molecules and peptides, natural products and synthetic molecules—and distinct pharmacology were designed [19,20,21,22,23,24]. Although targeting the KOR in drug discovery is very promising, the KOR is not devoid of detrimental side effects with receptor activation causing diuresis, dysphoria, sedation, psychotomimesis and anxiety in humans [15,16,17]. The small molecule with a morphinan scaffold, nalfurafine [25], and the peripherally acting peptide analogue, difelikephalin [26] (Figure 1), are two KOR agonists approved for clinical use as antipruritic drugs [27,28,29]. In addition, pain is a key clinical indication for KOR agonists, with experimental and clinical evidence that the KOR modulates pain processing in the CNS and PNS without the risk of physical dependence or abuse liability of MOR agonists [30,31,32,33].

Initially, KOR antagonists were widely used as pharmacological tools for studying the in vitro and in vivo actions upon KOR stimulation [19,20,21,28,34] The first selective KOR antagonists included ligands with a morphinan scaffold structure, i.e., nor-binaltorphimine (nor-BNI) [35], 5′-guanidinonaltrindole (5′-GNTI) [36] and the 4′-phenylpiperidine derivative, JDTic [37] (Figure 1). Preclinical studies showed that KOR inhibition or receptor depletion in the brain resulted in attenuation of depressive, anxiogenic affective and addictive-like behaviors, thus encouraging the development of selective KOR antagonists for the treatment of mood and addictive disorders. JDTic was the first selective KOR antagonist tested in humans for the treatment of cocaine abuse. However, clinical development of JDTic was terminated after modest cardiac abnormalities and an unfavorable brain-to-plasma concentration ratio, indicating poor CNS penetration [38]. Although preclinical and clinical data provide evidence on the therapeutic potential of KOR antagonists for CNS disorders, some peculiarities limit their usefulness [35,36]. The main issue with prototypical KOR antagonists (i.e., nor-BNI, 5′-GNTI and JDTic) is their exceptionally long duration of action, with multi-week blockades of the KOR activity following systemic administration after a single minimal dose. At higher doses, the antagonism may be further prolonged as demonstrated by studies performed with nor-BNI [34,39,40]. Delayed onset of KOR antagonism and side effects given by transient interaction with other opioid receptors, such as MOR antagonism after nor-BNI administration, have also been reported [41]. However, their abnormal long duration of action is, at present, the main concern about the feasibility of archetypical KOR antagonists. These findings have led to the development of short-acting KOR antagonists, including the pyrrolidine derivative, JNJ-67953964 (also known as LY2456302, CERC501 and aticaprant) [42], the quinolone, pyranyl and piperidine containing small molecule CYM-53003/BTRX-335140 [43] and different peptidic structures (i.e., zyklophin [44]) [19,20,21,23,45] (Figure 1). JNJ-67953964 is the first short-acting selective KOR antagonist shown to be safe in humans after oral administration and as a monotherapy for the treatment of major depressive disorders and substance use disorders. CYM-53003/BTRX-335140 is a further short-duration KOR antagonist, currently undergoing a phase 2 clinical trial for major depressive disorders [20,21].

Because of its therapeutic significance, the KOR is among the few GPCRs of which the X-ray crystal structures were determined both in inactive (Protein Data Bank, PDB-ID: 4DJH) [46] and active states (PDB-ID: 6B73 [47]). More recently, another structure of inactive-state KOR was solved with JDTic in complex with a Nb6 antibody (PDB-ID: 6VI4 [48]). The structure elucidation of the KOR and continued development of computational tools provide novel opportunities for computational modeling studies of receptor dynamics and for structure-based ligand discovery [49].

**Figure 2:**
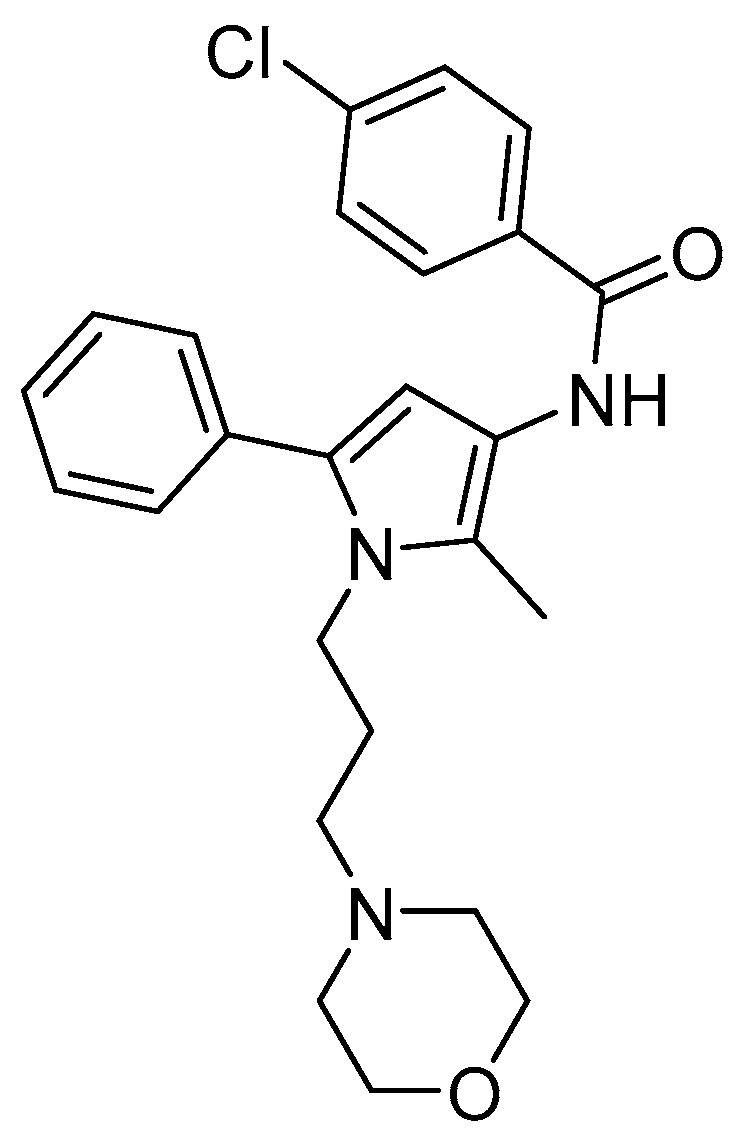
Structure of Compound A.

In the present study, we report on the in vitro, in vivo and in silico characterization of a new ligand as a KOR antagonist (Compound A, Figure 2). In an earlier study, Kaserer et al. [50] performed a 3D pharmacophore-based virtual screening campaign using several structure-based and ligand-based 3D query pharmacophores to discover novel ligands at the MOR. Compound A (as ‘compound 3’ in [50], Figure 2) was originally found as an MOR antagonist with very low binding affinity in the micromolar range to the human MOR. We have undertaken a comprehensive evaluation of Compound A, where experimental pharmacological (binding and functional in vitro assays and behavioral nociceptive models) and computational (in silico methods) approaches were combined, and established Compound A as a novel KOR antagonist with a structurally distinct scaffold compared to the so far known KOR ligands. We determined the binding mode of Compound A in complex with the KOR, as well as the MOR and DOR, and the structural determinants responsible for subtype selectivity of Compound A by conducting molecular docking and molecular dynamics (MD) simulations with subsequent dynophore (dynamic pharmacophore) generation of Compound A bound to the three classical opioid receptors.

## 2. Results and Discussion

### 2.1. Compound A Binds at the KOR with Increased Affinity vs. MOR, Lacks Specific Binding at the DOR, and Displays KOR Antagonism In Vitro

**Figure 3:**
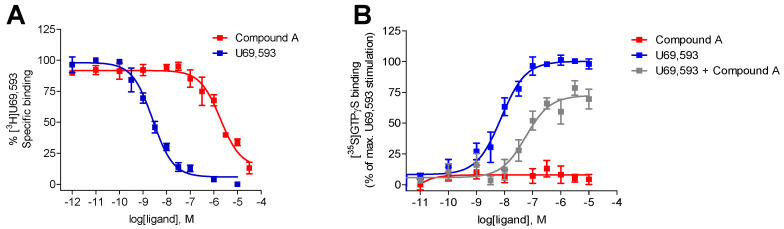
In vitro activity profile of Compound A at the human KOR. (**A**) Concentration-dependent inhibition by Compound A and U69,593 of [^3^H]U69,593 binding to membranes from CHO-hKOR cells determined in radioligand competitive binding assays. (**B**) Concentration-dependent stimulation of [^35^S]GTPγS binding by Compound A and U69,593, and effect of Compound A on U69,593-stimulated [^35^S]GTPγS binding to membranes from CHO-hKOR cells determined in the [^35^S]GTPγS binding assays. Values represent means ± SEM of at least 3 independent experiments performed in duplicate.

Whereas Compound A was previously described to interact with the human MOR and to exhibit antagonist properties, albeit with a very reduced binding affinity (inhibition constant as K_i_ value of 10.7 µM) [50] (Table 1), its receptor binding/selectivity and activation profile at the KOR and DOR were not reported. In this study, the binding of Compound A to the human KOR was evaluated using in vitro radioligand competitive binding assays with membrane preparations from Chinese hamster ovary cells stably expressing the human KOR (CHO-hKOR cells) and the specific KOR radioligand [^3^H]U69,593, according to the published procedure [51]. As shown in Figure 3A, Compound A produced a concentration-dependent inhibition of [^3^H]U69,593 binding displaying relatively moderate affinity at the human KOR (K_i_ = 1.35 µM), while the reference KOR ligand, U69,593 had a very high affinity in the low nanomolar range (Table 1). Additionally, competitive inhibition by Compound A of [^3^H]diprenorphine binding at the human DOR was assessed using in vitro radioligand binding assays with membranes from CHO-hDOR cells. Compound A displayed no substantial binding at the DOR at the concentration of 10 µM (% inhibition = 0.31 ± 6.52, *n* = 4) (Table 1). In the same assay, the r standard DOR ligand, naltrindole presented a very high affinity (K_i_ = 0.81 ± 0.04 nM) at the human DOR. Based on the current in vitro competition binding results, Compound A binds at the human KOR with increased affinity than to the MOR (ca. 8-fold), and it is devoid of specific binding at the DOR, therefore, presenting a preferential KOR selectivity profile.

Next, we have evaluated the in vitro functional activity of Compound A at the human KOR in the guanosine-5′-O-(3-[^35^S]thio)-triphosphate ([^35^S]GTPγS) binding assay, which measures KOR-mediated G protein activation upon ligand binding to the receptor. Previously, Compound A was reported as an MOR antagonist [48], but its antagonist potency was not determined because of its extremely low affinity at the MOR (Table 1) [50]. In this study, the [^35^S]GTPγS functional assay was performed with membranes from CHO cells, stably expressing the human KOR as described previously [51]. As shown in Figure 3B, Compound A did not increase the [^35^S]GTPγS binding in membranes from CHO-hKOR cells, indicating an antagonist profile at the KOR, in contrast to the high potency and stimulatory effect shown by the prototypical KOR agonist U69,593 (Table 1). Additional investigations established the antagonist properties of Compound A at the KOR, based on the rightwards shift (ca. 13-fold) in the U69,593 concentration–response curve in the presence of 10 μM of Compound A (Figure 3B), thus giving an antagonist equilibrium constant (K_e_) of 1.53 μM (Table 1). Our present results from the [^35^S]GTPγS functional assay establish Compound A as a new KOR ligand with antagonist properties in vitro. 

Compound A shows noticeable lower binding affinity and antagonist potency in the micromolar range to the KOR (Table 1) versus subnanomolar to nanomolar K_i_ and K_e_ values reported for known KOR antagonists (Table S1).

### 2.2. Subcutaneous Administration of Compound A Antagonized the KOR-Mediated Antinociception Induced by U50,488 in Mice

**Figure 4:**
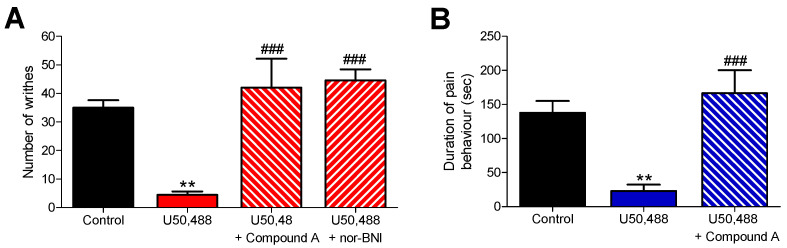
In vivo KOR antagonism of Compound A. Antagonism of U50,488-induced antinociception in mice by Compound A after s.c. administration in (**A**) the acetic acid-induced writing assay and (**B**) the formalin test. (**A**) In the writhing assay, groups of mice received s.c. control (vehicle), U50,488 (2 mg/kg) or were s.c. pre-treated with Compound A (10 mg/kg, −15 min) or nor-BNI (10 mg/kg, −24 h) before U50,488, and the number of writhes were counted for 10 min. Values represent means ± SEM (*n* = 5–6 mice per group). (**B**) In the formalin test, groups of mice received s.c. control (vehicle), U50,488 (1 mg/kg) or were s.c. pre-treated with Compound A (10 mg/kg, −15 min) before U50,488, and the duration of pain behavior (time spent licking, biting, lifting and flinching the formalin-injected paw) was counted for 15 min, starting 15 min after formalin injection. Values represent means ± SEM (*n* = 6–8 mice per group). ** *p* < 0.01 vs. control (vehicle) group; ^###^ *p* < 0.001 vs. U50.488-treated group; one-way ANOVA with Tukey’s post hoc test.

Based on the in vitro results, the KOR antagonist activity of Compound A was evaluated in vivo in mouse models of visceral pain (acetic acid-induced writhing assay) and inflammatory pain (the formalin test), according to previously described procedures [51,52]. To this aim, Compound A, administered to mice subcutaneously (s.c.), was assessed for its capability to antagonize the antinociceptive effect produced by the typical KOR agonist U50,488 in both pain models (Figure 4). When Compound A (10 mg/kg, 22.7 µmol/kg) was injected 15 min prior to U50,488 (2 mg/kg, s.c.) in the formalin assay, a significant and complete reversal of U50,488-induced inhibition of writhing behavior was measured (Figure 4A), demonstrating a KOR-mediate mechanism. Similarly, pretreatment of mice with the standard KOR antagonist nor-BNI (10 mg/kg, 13.6 µmol/kg, s.c.) for 24 h before U50,488 blocked the antinociceptive effect of the KOR agonist in the writhing assay (Figure 4A). Compound A was about twofold less potent than nor-BNI as a KOR antagonist in vivo. We further established that pretreatment of mice with Compound A (10 mg/kg, 22.7 µmol/kg s.c.) significantly antagonized the reduction of pain behaviors caused by U50,488 (1 mg/kg, s.c.) during the inflammatory phase of the formalin test, quantified by an increase in the amount of time each animal spent licking, biting, lifting and flinching the formalin-injected paw (Figure 4B). Thus, we show in two pain models that Compound A after s.c. administration to mice behaves as a KOR antagonist in vivo.

### 2.3. Modeling Inactive KOR Based on X-ray Crystal Structure 4DJH including Refinement of Transmembrane Helix 1

A comparison of the three inactive state X-ray crystal structures of the KOR, MOR and DOR (PDB-IDs: 4DJH [44], 4DKL [53], and 4N6H [54], respectively) reveals an overall similar global fold with the exception of the extracellular half of transmembrane helix (TM) 1. Although the MOR and DOR share a similar conformation of this helix, the extracellular half of TM1 is strongly bent outwards at the KOR (8.3 Å between the KOR and DOR, and 7.3 Å between the KOR and MOR; measured between Cα of residue 1.30 at the top of TM1 for each receptor pair). The inactive structure of the KOR (PDB-ID: 4DJH) was crystallized as a parallel dimer with the dimer interface consisting of TM1, 2 and 8 [46], i.e., the KOR TM1 conformation is likely influenced by the second receptor within the cell unit of the crystal structure. To ensure consistency with the global fold of the opioid receptors under investigation, and to remove potential artefacts from dimer crystallization, we remodeled the TM1 region of the KOR crystal structure. Thus, we modeled the upper half of TM1 (S55-V72) based on the DOR crystal structure (PDB-ID: 4N6H [54], 66.7% similarity within this region) as described in the Section 3. We thus obtained coordinates for the KOR that are based on the crystal structure but include a refined upper TM1 region.

### 2.4. Docking Reveals Stabilizing Interactions between the Chlorophenyl Moiety of Compound A and the KOR Responsible for the Highest Subtype Affinity

**Figure 5:**
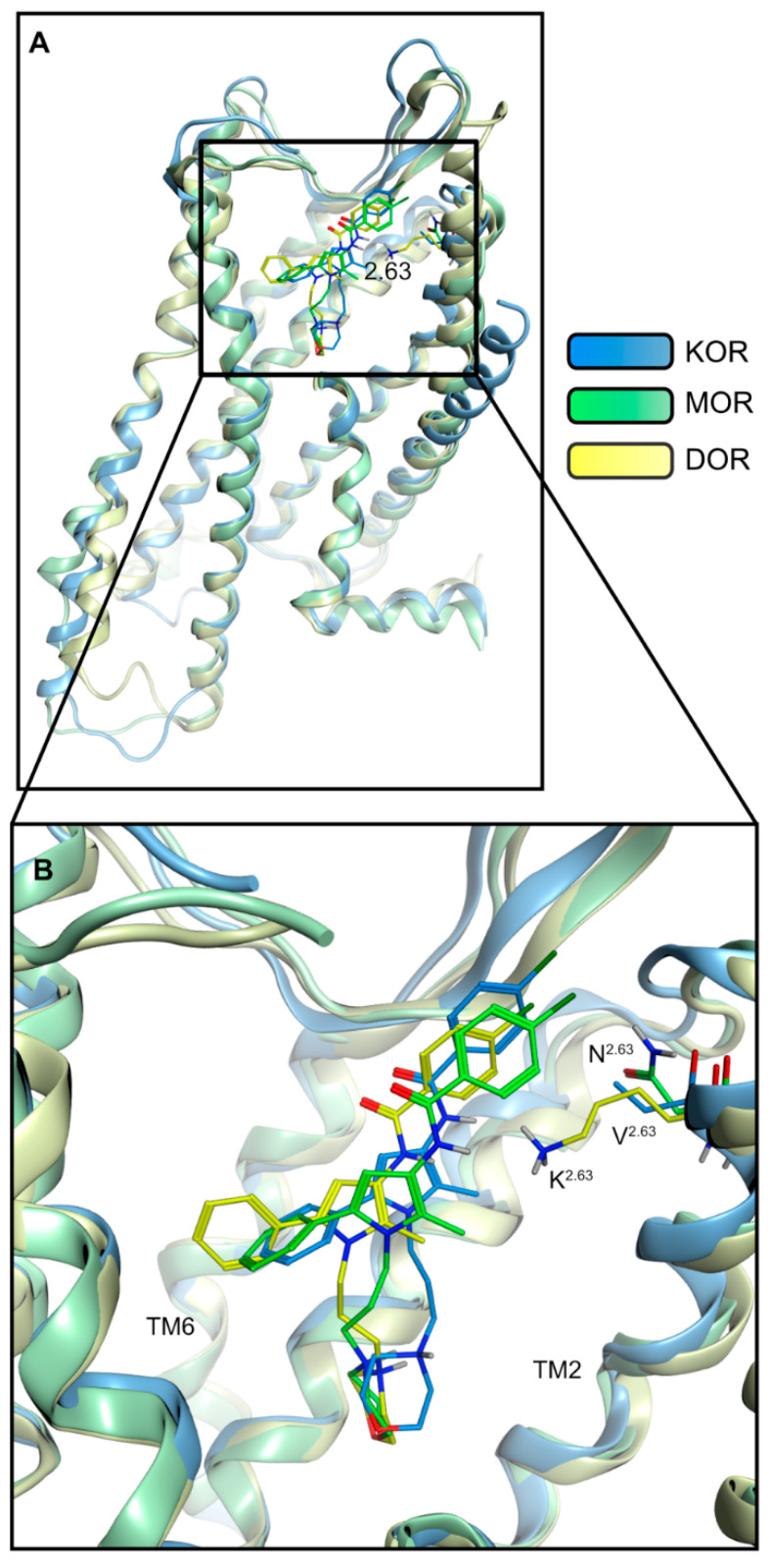
Binding mode of Compound A at the KOR, MOR and DOR. (**A**) Compound A binds within the orthosteric pocket in the extracellular half of the receptors. (**B**) Compound A shares an overall similar orientation within the receptors. The residues 7.28–7.41 are not shown for better visualization.

To investigate the mechanistic determinants of the different experimentally determined binding affinities of Compound A to the classical opioid receptor subtypes, we performed docking experiments of Compound A into the prepared crystal structures of the MOR (PDB-ID: 4DKL [53]), DOR (PDB-ID: 4N6H [54]) and the KOR model (based on PDB-ID: 4DJH [46]). We docked Compound A into the orthosteric binding pocket of the three opioid receptors as described in the Section 3. In our docking experiments, the morpholine group of Compound A deeply protrudes into the orthosteric binding pocket, establishing an ionic interaction between the positively charged nitrogen of the morpholine moiety and the carboxylate of D^3.32^ (KOR: D138^3.32^, MOR: D149^3.32^ and DOR: D128^3.32^, superscripts denote Ballesteros–Weinstein numbering [55]) that is known to be crucial for ligand binding [46,47,56,57]. The phenyl group of Compound A points towards TM5 and TM6, establishing extensive lipophilic contacts to residues within TM3, TM5 and TM6, while the chlorophenyl moiety further extends toward the extracellular side and points toward TM2 and TM3 (Figure 5). The non-conserved residue K108^2.63^ in TM2 in the DOR extends further into the binding pocket than the respective residues in the KOR (V118^2.63^) and MOR (N129^2.63^), causing a shift of Compound A toward TM5 and TM6 in the DOR compared to the KOR and MOR.

**Figure 6:**
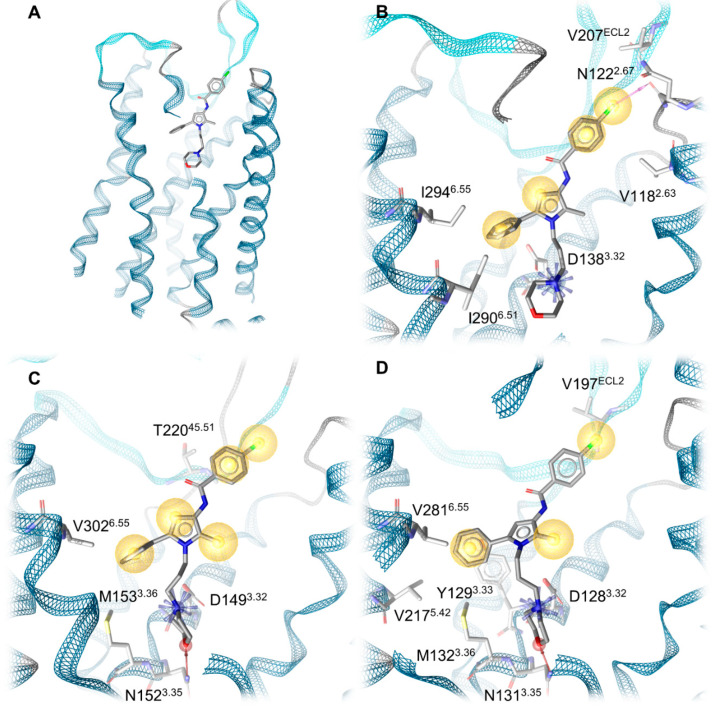
Protein–ligand interactions. (**A**) Global view of Compound A bound to the KOR. (**B**) Protein–ligand interactions at the KOR. (**C**) Protein–ligand interactions at the MOR. (**D**) Protein–ligand interactions at the DOR. Blue stars indicate positive charges, yellow spheres lipophilic contacts, pink arrows halogen bond donors and red arrows hydrogen bond acceptors. The residues 313–319 in the KOR, 314–327 in the MOR and 276–279 as well as 296–307 in the DOR are not shown for better visualization.

Development of 3D pharmacophores of the opioid receptor–Compound A complexes reveals a different number of interactions between the chlorophenyl moiety and the subpocket (residues 2.63, 2.67, 2.68, 2.69, 3.29, part of extracellular loop, ECL2) that accommodates this chlorophenyl moiety (Figure 6). The KOR establishes the most interactions, specifically three, followed by two interactions in the MOR and only one in the DOR (Table 2). In the KOR, the chlorophenyl moiety binds to the subpocket via two hydrophobic contacts (V188^2.63^, V207^ECL2^) and a halogen bond towards N122^2.67^. These interactions likely improve the affinity of Compound A at the KOR (K_i_ = 1.35 µM, Table 1). Conversely, at the MOR, the chlorophenyl moiety of Compound A is only stabilized by two hydrophobic contacts with a threonine side chain methyl group (T220^45.51/ECL2^). This likely causes the decreased affinity of Compound A towards the MOR (K_i_ = 10.7 µM, Table 1) compared to the KOR. In the DOR, the positively charged K108^2.63^ deeply points into the subpocket towards the chlorophenyl moiety of Compound A. The close proximity between the chlorophenyl moiety of Compound A and K108^2.63^ (3.5 Å, measured between the primary amine nitrogen of K108^2.63^ and the closest carbon of the chlorophenyl of Compound A) likely contributes to the absence of binding of Compound A in concentrations up to 10 µM to the DOR. Additionally, the phenyl ring of the chlorophenyl moiety of Compound A does not participate in any interactions with the DOR, in contrast to the KOR and MOR. Only the chlorine forms a hydrophobic contact with V197^ECL2^. Table 2 shows all protein–ligand interactions from our docking experiments for comparison.

**Figure 7:**
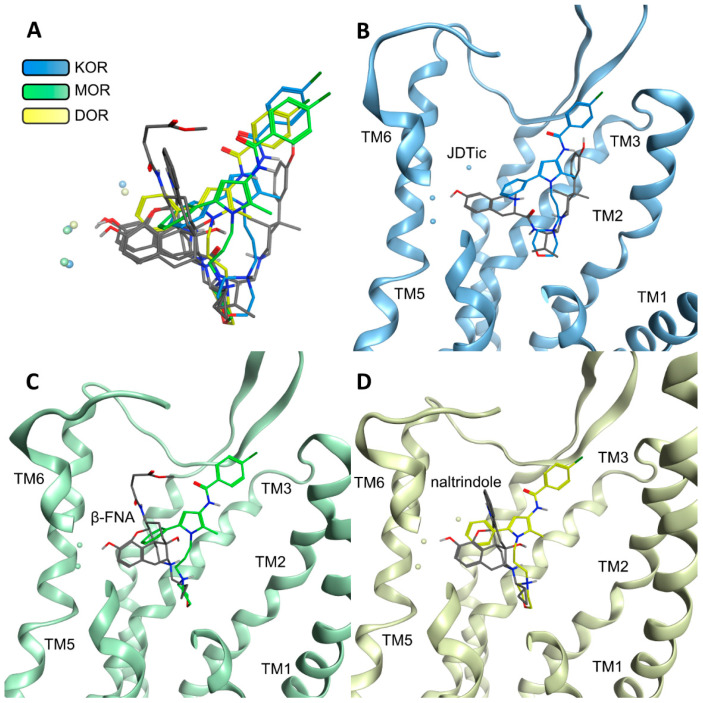
Comparison of the binding modes of Compound A and the binding modes of the co-crystallized ligands. (**A**) Overlay of all compared ligands. Co-crystallized ligands are depicted in gray. (**B**) JDTic and Compound A in the KOR. (**C**) β-FNA and Compound A in the MOR. (**D**) Naltrindole and Compound A in the DOR. Water molecules are shown as spheres without hydrogens.

Compound A is a weak opioid receptor binder with affinity values in the micromolar range (Table 1). To rationalize the low binding affinity, we performed a comparison of Compound A and the co-crystallized high-affinity ligands from the inactive crystal structures used for docking (KOR: JDTic, MOR: β-FNA, and DOR: naltrindole) in complex with the opioid receptors (Figure 7). This comparison reveals that the co-crystallized ligands are shifted toward TM5/TM6 in their corresponding complexes with respect to Compound A. Their bulky ring systems, containing a phenol group in all three ligands, are the moieties closest to TM5/TM6 and the hydroxyl groups of the phenol moieties take part in water-mediated hydrogen bonds connecting the co-crystallized ligands to TM3/TM5 and TM6 (KOR: Y139^3.33^, K227^5.39^ and H291^6.52^; MOR: K235^5.39^ and H299^6.52^, DOR: Y129^3.33^, K214^5.39^and H278^6.52^). The phenyl group of Compound A does not participate in water-mediated hydrogen bonds as it does not point as far toward TM5/TM6 and contains no functional group capable of hydrogen bonding. A phenol moiety that interacts with TM5 is a common feature for opioids [47] and its absence in Compound A likely contributes to its low binding affinity. Additionally, it was previously reported that the presence of a phenol group is more important in the MOR than in the KOR [47], which is in accordance with the higher affinity of Compound A to the KOR compared to the MOR.

### 2.5. Molecular Dynamics Simulations Reveal the Most Durable and Frequent Interaction Pattern of the Chlorophenyl Moiety of Compound A in the KOR Complex

**Figure 8:**
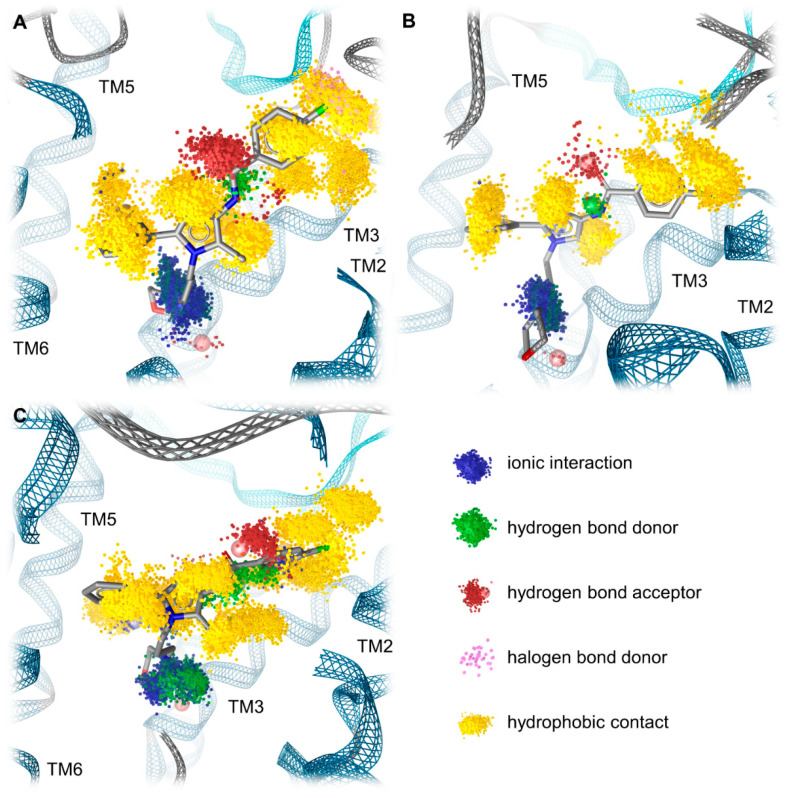
Dynamic 3D pharmacophores (dynophores) of Compound A–opioid receptor complexes. (**A**) KOR. (**B**) MOR. (**C**) DOR. Chemical feature clouds refer to interactions occurring over simulation time.

To further investigate our static binding hypotheses, we performed molecular dynamics (MD) simulations and developed dynamic 3D pharmacophore models (dynophores [58]). The dynamic evaluation confirms our static hypothesis as it reveals the most durable and frequent interactions for the chlorophenyl moiety of Compound A in the KOR complex. The chlorophenyl moiety of Compound A participates in hydrophobic contacts in 99.1% and 98.4% (values for the chlorine and the chlorophenyl plane, respectively) of the simulation time (Table 3). Additionally, the chlorophenyl moiety is stabilized by a halogen bond in 13.3% of the simulation time that is not present in the complexes with the MOR and DOR (Table 3). These interaction patterns of the chlorophenyl moiety likely contribute to the affinity of Compound A measured at the KOR being the highest out of the three investigated complexes. In complex with the MOR, Compound A is also stabilized by hydrophobic contacts in the vast majority of the simulation time (99.8% and 99.2% for the chlorine and the chlorophenyl plane, respectively), but lacks the additional stabilization by halogen bonding (Table 3). The missing halogen bond appears to decrease the affinity of Compound A towards the MOR. At the DOR, Compound A does not only lack the halogen bond but also engages in less frequent hydrophobic contacts in total (100% and 83.2% for the chlorine and the chlorophenyl plane, respectively) compared to the KOR and MOR (Table 3), which likely explains the experimentally measured absence of affinity at the DOR. The difference in the stabilization of the chlorophenyl moiety at the opioid receptor complexes by hydrophobic contacts is even more pronounced when considering the total number of hydrophobic contacts. In the KOR and MOR, a similar absolute number of hydrophobic contacts within the whole trajectories was counted (KOR: 4730, MOR: 5243), while there were fewer contacts detected in the DOR complex (3277, corresponding to 69.3% of the contact number counted at the KOR). This discrepancy further rationalizes the experimentally measured differences in the binding affinity of Compound A towards the three opioid receptor subtypes. The dynamic pharmacophores of Compound A within the opioid receptor complexes are shown in Figure 8.

In order to address the protein and ligand conformational stability, we performed root mean square deviation (RMSD) calculations for the opioid receptors and Compound A, as well as heavy atom root mean square fluctuation (RMSF) calculations for the receptors over the simulation time. The information is presented in the Supplementary Materials (Figures S1–S9). To track the receptor compactness along the simulations, we calculated the radius of gyration of the receptors over the simulation time (Figures S10–S12). The radius of gyration values remains stable over the trajectory, indicating steady receptor compactness. In order to monitor the correct protein folding over the course of the MD simulations, we calculated the solvent accessible surface areas (SASA) values of the receptors (Figures S13–S15). No strong increase in the SASA values was found, indicating that no protein unfolding processes could be observed.

### 2.6. Compound A Shows Favorable Physicochemical Properties and Is a CNS Penetrant KOR Antagonist

Evaluation of pharmacokinetic properties represents a key feature in today’s drug discovery, particularly in predicting response profiles in vivo of bioactive molecules [59,60]. We have calculated and compared the partition coefficients (*c*logP) and distribution coefficients at pH 7.4 (*c*logD_7.4_) of Compound A and various small molecules KOR antagonists (Table 4). In general, compounds with higher hydrophobicity, i.e., larger *c*logP and *c*logD_7.4_ values, are expected to readily cross the blood–brain barrier [61]. According to the *c*logP and the *c*logD_7.4_, Compound A shows favorable physicochemical features and a better capability to enter the CNS compared to the known KOR antagonists, that show increased hydrophilicity at physiological pH.

Further calculations based on chemical properties of Compound A, including ADMET properties and bioavailability (BOILED-Egg plot [63]), are presented in the Supplementary Materials (Figures S16 and S17, Table S2). The calculations were performed using the open access SwissADME web tool [64]. Compound A was predicted to have high gastrointestinal absorption and to readily pass the blood–brain barrier but also as a permeability–glycoprotein substrate.

## 3. Materials and Methods

### 3.1. Chemicals and Reagents

Radioligands [^3^H]U69,593 (49.3 Ci/mmol), [^3^H]diprenorphine (33.9 Ci/mmol) and [^35^S]GTPγS (1250 Ci/mmol) were purchased from PerkinElmer (Boston, MA, USA). Guanosine diphosphate (GDP), GTPγS, U69,593, U50,488, diprenorphine, tris(hydroxymethyl) aminomethane (Tris), 2-[4-(2-hydroxyethyl)piperazin-1-yl]ethanesulfonic acid (HEPES), bovine serum albumin (BSA), formalin, nor-BNI and cell culture media and supplements were obtained from Sigma-Aldrich Chemicals (St. Louis, MO, USA). All other chemicals were of analytical grade and obtained from standard commercial sources. Compound A was obtained from Maybridge Chemical Co., Ltd. (Cornwall, UK) as in [50], and was prepared as 1 mM stock in 0.5% acetic acid solution and further diluted to working concentrations in the appropriate medium.

### 3.2. Cell Cultures and Cell Membrane Preparation

CHO cells stably expressing the human opioid receptors (CHO-hKOR and CHO-hDOR cell lines) were kindly provided by Lawrence Toll (SRI International, Menlo Park, CA). CHO-hKOR cells were grown at 37 °C in Dulbecco’s Modified Eagle’s Medium (DMEM) culture medium and supplemented with 10% fetal bovine serum (FBS), 0.1% penicillin/streptomycin, 2 mM L-glutamine and 0.4 mg/mL geneticin (G418). CHO-hDOR cells were grown at 37 °C in DMEM/Ham’s F12 culture medium and supplemented with 10% FBS, 0.1% penicillin/streptomycin, 2 mM L-glutamine and 0.4 mg/mL geneticin (G418). All cell cultures were maintained in a humidified atmosphere of 95% air and 5% CO_2_. Membranes from CHO-hOR cells were prepared as previously described [51]. Briefly, CHO-hOR cells grown at confluence were removed from the culture plates by scraping, homogenized in 50 mM Tris-HCl buffer (pH 7.7) using a Dounce glass homogenizer, then centrifuged once and washed by an additional centrifugation at 27,000× *g* for 15 min at 4 °C. The final pellet was resuspended in 50 mM Tris-HCl buffer (pH 7.7) and stored at −80 °C until use. Protein content of cell membrane preparations was determined by the method of Bradford using BSA as the standard [65].

### 3.3. Competitive Radioligand Binding Assays

In vitro binding assays were conducted on human opioid receptors stably transfected into CHO cells according to the published procedures [51]. Assays were performed in 50 mM Tris-HCl buffer (pH 7.4) in a final volume of 1 mL. Cell membranes (20 µg) were incubated with various concentrations of test compounds of [^3^H]U69,593 (0.4 nM) or [^3^H]diprenorphine (0.2 nM) for labeling KOR or DOR, respectively, for 60 min at 25 °C. Non-specific binding was determined using 10 µM U69,593 or 1 µM diprenorphine. After incubation, reactions were terminated by rapid filtration through Whatman GF/C glass fiber filters. Filters were washed three times with 5 mL of ice-cold 50 mM Tris-HCl buffer (pH 7.4) using a Brandel M24R cell harvester (Brandel, Gaithersburg, MD, USA). Radioactivity retained on the filters was counted by liquid scintillation counting using a Beckman Coulter LS6500 (Beckman Coulter Inc., Fullerton, CA, USA). Inhibition constant (K_i_, nM) values were determined by the method of Cheng and Prusoff [66] from concentration–response curves by nonlinear regression analysis using the GraphPad Prism 5.0 Software (GraphPad Prism Software Inc., San Diego, CA, USA). All experiments were performed in duplicate and repeated at least three times with independently prepared samples.

### 3.4. [^35^S]GTPγS Binding Assays

Binding of [^35^S]GTPγS to membranes from CHO stably expressing the human KOR was conducted according to the published procedure [51]. Cell membranes (15 µg) in 20 mM HEPES buffer (pH 7.4) supplemented with 10 mM MgCl_2_ and 100 mM NaCl were incubated with 0.05 nM [^35^S]GTPγS, 10 µM GDP and various concentrations of test compounds in a final volume of 1 mL for 60 min at 25 °C. Non-specific binding was determined using 10 µM GTPγS, and the basal binding was determined in the absence of test ligand. Samples were filtered over Whatman GF/B glass fiber filters and counted as described for competitive radioligand binding assays. The increase in [^35^S]GTPγS binding above the basal activity was used to determine potency (EC_50_, in nM) and efficacy (as % stimulation of maximum stimulation with respect to the reference KOR full agonist, U69,593, which was set as 100%) from concentration–response curves by nonlinear regression analysis using the GraphPad Prism 5.0 Software (GraphPad Prism Software Inc., San Diego, CA, USA). To determine the KOR antagonist potency of Compound A, the Schild analysis was performed, where a concentration–response curve for U69,593 was obtained by assessing the [^35^S]GTPγS binding to CHO-hKOR cell membranes in the presence or absence of Compound A. The equilibrium dissociation constant (K_e_) was calculated from the equation K_e_ = [a]/(D − 1), where “a” is the concentration of antagonist, and DR is the ratio of EC_50_ values of U69,593 in the presence and absence of Compound A. All experiments were performed in duplicate and repeated at least three times with independently prepared samples.

### 3.5. Animals and Drug Administration

Experiments were performed in male CD1 mice (8–10 weeks old, 30–35 g body weight) purchased from Janvier Labs (Le Genest-Saint-Isle, France). All animal care and experimental procedures were in accordance with the ethical guidelines for the animal welfare standards of the European Communities Council Directive (2010/63/EU) and were approved by the Committee of Animal Care of the Austrian Federal Ministry of Science and Research. Mice were group-housed in a temperature-controlled specific pathogen free room with a 12 h light/dark cycle and with free access to food and water. U50,488 and nor-BNI were prepared in sterile physiological saline (0.9%). Compound A was prepared in 1% acetic acid solution in sterile physiological saline (0.9%). Test compounds or vehicle (saline) were administered s.c. in a volume of 10 µL/g body weight.

### 3.6. Acetic Acid-Induced Writhing Assay

Writhing was induced in mice by intraperitoneal (i.p.) injection of a 0.6% acetic acid aqueous solution as described previously [51]. Following a habituation period of 15 min to individual transparent observation chambers, mice were s.c. administered U50,488 (2 mg/kg) or control (vehicle), and after 25 min (5 min prior to testing) each animal received i.p. injection of acetic acid solution. The number of writhes was counted during a 10 min observation period. For the antagonism study, Compound A (10 mg/kg) and nor-BNI (10 mg/kg) were s.c. administered 15 min and 24 h, respectively, before U50,488 (2 mg/kg, s.c.), and writhing behavior was assessed as described above.

### 3.7. Formalin Test

The formalin test was performed as described previously [52]. Following a habituation period of 15 min to individual transparent observation chambers, mice were s.c. administered U50,488 (1 mg/kg) or control (vehicle), 5 min prior injection of 20 µL of 5% formalin aqueous solution to the plantar surface of the right hindpaw. The time (in s) each animal spent licking, biting, lifting and flinching the formalin-injected paw (pain behavior) was recorded in 5 min intervals between 15 and 30 min after the injection of formalin (Phase II reaction). For the antagonism study, Compound A (10 mg/kg) was s.c. administered 15 min before U50,488 (1 mg/kg, s.c.), and pain behavior was assessed as described above.

### 3.8. Data and Statistical Analysis

Experimental data were graphically processed and statistically analyzed using the GraphPad Prism Software (GraphPad Prism Software Inc., San Diego, CA, USA) and are presented as means ± SEM. Data were statistically evaluated using one-way ANOVA with Tukey’s post hoc test for multiple comparisons between the treatment groups, with significance set at *p* < 0.05.

### 3.9. Protein Preparation

The inactive-state X-ray crystal structures of the three opioid receptors were retrieved from the protein data bank (PDB [67]) with PDB-ID: 4DJH for KOR [46], PDB-ID: 4DKL for MOR [53] and PDB-ID: 4N6H for DOR [53]). The structure preparation was carried out in a Molecular Operating Environment (MOE v2020.0901) [68] and focused on the chain with the better resolution out of the two chains in the KOR dimer (PDB-ID: 4DJH). Firstly, we deleted the unresolved parts of the chains as well as fusion proteins (T4 lysozyme in KOR and MOR, b562RIL (BRIL) in DOR). To restore the human receptors to wild-type we used the human wild-type sequence obtained from the UniProt-Databank [69] to revert thermostabilizing mutations in the DOR and KOR (human DOR: P41143, S37P; human KOR: P41145, L135I). The PDB-ID: 4DKL (MOR, [53]) encodes the mouse MOR. Thus, we reverted four mouse-specific residues in the MOR to the human wild-type MOR residues using the UniProt-ID: P35372 (V68I, N139T, V189I, I308V). Broken loops due to unresolved parts of ECL3 and ICL3 of the KOR as well as of ICL3 of the MOR were modeled using the loop modeler function while missing side chain atoms were generated using the protein builder, both integrated into MOE. Subsequently, Ramachandran outliers [70] and atom clashes were resolved using energy minimization with the OPLS-AA force field [71].

Due to the dimerization of the KOR chains within a cell unit (PDB-ID: 4DJH), the extracellular portion of TM1 is bent outwards along the receptor’s longitudinal axis. Hence, we restored the global fold of the KOR-TM1 structure during the protein preparation to achieve a conformation comparable to the global folds of the MOR and DOR. For this purpose, a homology model of the upper half of the KOR-TM1 (S55-V72 according to UniProt-ID P41145) was built using the homology modeling tool implemented in MOE (v2020.0901) [68] with the DOR (PDB-ID: 4N6H) serving as a template. Within the homology model generation ten intermediate models were built at 300 K using the OPLS-AA force field [71] and scored according their electrostatic solvation energy [72]. The best-scored model was chosen for further geometric refinement yielding in the final model used in this study. The homology model was subsequently fused to the KOR inactive X-ray crystal structure (PDB-ID: 4DJH). The geometric properties of the TM1 homology model (fused to the KOR X-ray crystal structure) and surrounding residues were again optimized using energy minimization with the OPLS-AA force field [71]. Furthermore, the conformations of neighboring Y320^7.46^ and Q115^2.60^ were aligned according to the respective conformations in the DOR (Y308^7.43^, Q105^2.60^), using the rotamer tool within MOE v2020.0901 [68].

At the MOR inactive crystal structure (PDB-ID: 4DKL), the residue Y130^2.64^ adopts a conformation bend towards the TM1, which is not comparable to the DOR crystal structure (PDB-ID: 4N6H) and our KOR model. We surmise a single missing water molecule in the TM1 and TM2 region in the MOR crystal structure responsible for the conformation shift as the OH group in the corresponding Y109^2.64^ establishes a water-mediated hydrogen bond to the hydroxyl group in Y56^1.39^ at the high-resolution DOR crystal structure, which cannot be seen in the lower resolution MOR crystal structure. As we assume a similar water-mediated hydrogen bond in the MOR, supported by a weak electron density in the MOR structure that likely corresponds to a water molecule, we adjusted the orientation of the Y130^2.64^ side chain in the MOR manually, according to the respective orientation in the DOR (Y109^2.64^).

The protonate 3D function [73] implemented in MOE (v2020.0901) [68] was used to protonate all three opioid structures at pH 7 and temperature of 300 K.

All selected X-ray crystal structures contain some water molecules within the binding site. Only the water molecules HOH1303, HOH1307 and HOH1311 in case of the KOR, HOH718 and HOH719 in case of the MOR, and HOH1323, HOH1324 and HOH1336 in case of the DOR were retained for subsequent docking and MD simulations as they participate in water mediated interactions between the cocrystallized ligands and protein residues that are known to be involved in ligand binding and selectivity (KOR: K227^5.39^, H291^6.52^ and Y139^3.33^ [74]; MOR: K235^5.39^ and H299^6.52^ [75]; DOR: H278^6.52^, K214^5.39^and Y129^3.33^ [76,77,78].

### 3.10. Protein-Ligand Docking Study

Corina v3.00 [79,80] was used to generate the 3D conformation of Compound A used for docking. The protonate 3D function [73] implemented in MOE (v2020.0901) [68] was conducted to protonate Compound A at a pH of 7 and a temperature of 300 K. Subsequent docking of Compound A into the orthosteric pocket of the opioid receptors was performed using GOLD v5.2 [81]. A 20 Å sphere with the side chain carboxylate carbon atom of D^3.32^ (KOR: D138^3.32^, MOR: D149^3.32^ and DOR: D128^3.32^) as its center defined the binding site of the receptors, which was limited to the solvent-accessible surface. For each opioid receptor structure, a total number of 30 genetic algorithm runs were performed, yielding diverse solutions (i.e., more than 1.5 Å RMSD between the binding hypotheses of each performed docking process). The search efficiency was set at 100%. To account for the physiological flexibility of pyramidal nitrogen atoms, these atoms were allowed to flip within the ligand throughout the docking process. All obtained docking poses were scored according the GoldScore docking function [82,83] implemented in GOLD v5.2 [81]. An ionic interaction between the carboxylate of D^3.32^ at the opioid receptors and a protonatable nitrogen of the ligand is known to be crucial for ligand binding [44,45,54,55]. Thus, we set a constraint of a maximum distance of 5.5 Å between the protonatable morpholine nitrogen of Compound A and the γC-atom of D^3.32^.

After docking, the MMFF94 force field [84,85,86,87,88] incorporated in LigandScout v4.4.3 [89,90] was conducted to minimize the energy of the obtained binding hypotheses within the protein environment. The binding poses of Compound A in complex with the MOR, DOR and KOR were visually inspected and filtered according to the position of the positively charged morpholine nitrogen of Compound A within the receptor, essential for the opioid receptor activity [46,47,56,57], as well as the stabilization of Compound A via hydrophobic contacts to the receptors after generating 3D pharmacophores using LigandScout [89,90]. Hydrophobic contacts of Compound A towards TM2/TM3 region were preferred as they were already described for other non-morphinan antagonist, JDTic [46], and tetrapeptide DIPP-NH_2_ [56].

### 3.11. Molecular Dynamics Simulations and Analysis

We performed three MD simulations of 100 ns length for each of the receptor–ligand complexes. We used Maestro v2020-4 [91] for system setup, OPLS 2005 force field [92,93] for system parametrization and Desmond v2020-4 [94] for performance of the MD simulations. For each system, we positioned the protein in a cubic box with 10 Å padding each side to the protein surface. A POPC (1-palmitoyl-2-oleoylphosphatidylcholine) bilayer was used to mimic the physiological membranes, and the proteins were embedded in these membranes according the OPM database [95] (PDB-ID: 4DJH for the KOR, 4DKL for the MOR, and 4N6H for the DOR). The remaining space in the box was subsequently filled with TIP4P water molecules [96] and ions (Na^+^, Cl^−^), leading to an isotonic solution (0.15 M NaCl). During the simulations, a constant number of particles, pressure (1.01325 bar), and a constant temperature (300 K) were maintained (NPT ensemble). The simulations were run for 100 ns each, resulting in 1000 distinct ligand–receptor conformations sampled per simulation. Centering of the protein and the alignment of the respective trajectories onto the backbone-heavy atoms of the first protein conformation sampled during the simulation were performed using VMD v1.9.3 [95].

For subsequent MD simulation analysis, we generated dynamic pharmacophores of Compound A over the simulation time using the Dynophore software (version 0.1, Gerhard Wolber, Berlin, Germany) [58,97]. Only interactions occurring for a minimum of 5% of the simulation time were considered for evaluation of MD simulations. Root mean square deviation (RMSD) and solvent accessible surface area (SASA) calculations of MD simulations were conducted using VMD v1.9.3 [98]. Root mean square fluctuation (RMSF) and radius of gyration calculations were performed using Maestro v2020-4 [91].

## 4. Conclusions

In conclusion, we reported on a comprehensive study aided by in vitro and in vivo assays and computational techniques where Compound A was characterized as a novel KOR antagonist. Our interesting observations from radioligand competitive binding and functional in vitro assays revealed Compound A to bind at the KOR, albeit with moderate affinity (in low micromolar range), but with increased affinity than to the MOR and to lack specific binding at the DOR, thus displaying a favorable KOR selectivity profile. Additionally, behavioral investigations in mice established the in vivo KOR antagonist properties of Compound A after s.c. administration, based on its ability to effectively reverse the antinociceptive effects of the prototypical KOR agonist, U50,488, in two pain models, the writhing assay and the formalin test.

At the in silico level, we performed molecular docking and MD simulations using the inactive state crystal structures of the KOR, MOR and DOR, in order to further assess the structural determinants responsible for receptor subtype selectivity of Compound A. Our molecular docking study on Compound A into the orthosteric site pocket of KOR, MOR and DOR revealed distinct interaction patterns (pharmacophores) between the chlorophenyl moiety of Compound A and each opioid receptor subtype, which well correlated with the affinity (KOR > MOR >>>> DOR) of Compound A determined experimentally. The structure of the KOR exhibits two hydrophobic contacts (V207^ECL2^, V118^2.63^) and one halogen bond (N122^2.67^) to Compound A, correlating with the highest binding affinity experimentally measured. The structure of the MOR exhibits only two contacts with the chlorophenyl moiety of Compound A (via T220^ECL2^), leading to a binding affinity approximately one order of magnitude less compared to Compound A’s affinity at the MOR. At the DOR, only the chloride is stabilized in a hydrophobic contact while the chlorophenyl plane does not take part in any interactions. This sparser interaction pattern, together with the bulky side chain K108^2.63^ pointing into the binding site, thereby shifting Compound A out of the subpocket, likely contributes to the lack of binding of Compound A to the DOR, as determined experimentally. Furthermore, MD simulations of the opioid receptor–Compound A complexes revealed the strongest stabilization of Compound A’s chlorophenyl moiety at the KOR with frequent hydrophobic contacts supported by halogen bonding. Although the ligand–MOR complex lacks the halogen bonding, the number of hydrophobic contacts at the DOR is decreased. Thus, the MD simulations confirmed our results obtained by docking.

Notably, Compound A shows a good capability to enter the CNS (based on the *c*logP and *c*logD_7.4_), and it has a structurally distinct scaffold compared to the so far known KOR ligands (Figure 1 and Figure 2). Although Compound A interacts with the KOR relatively weakly, this new chemotype shows promising KOR antagonist properties in vitro and in vivo. Thus, Compound A represents a valuable starting point for chemical optimization toward the development of innovative ligands as potential therapeutics for human conditions where the kappa opioid system has a key function.

## Figures and Tables

**Table 1:** In vitro binding affinities and functional activities of Compound A at the human KOR.

	Opioid Receptor Binding (K_i_, µM) ^a^			[^35^S]GTPγS Binding, KOR ^b^		
	KOR	MOR	DOR	EC_50_ (µM)	% stim.	K_e_ (µM)
Compound A	1.35 ± 0.32	10.7 ± 4.7 ^c^	- ^d^	- ^e^	- ^e^	1.53 ± 0.38
U69,593	0.0019 ± 0.0004	n.d.	n.d.	0.011 ± 0.004	100	n.a.

^a^ Determined in radioligand competitive binding assays using membranes of CHO cell stably expressing the human KOR (CHO-hKOR). ^b^ Determined in the [^35^S]GTPγS binding assay with membranes of CHO-hKOR cells. Efficacy (% stim.) is expressed as percentage stimulation relative to the maximum effect of the KOR full agonist U69,593 (as 100%). ^c^ Data from [50]. ^d^ No specific binding was detected at 10 µM in the radioligand binding assays using CHO-hDOR cell membranes. ^e^ No stimulation up to 10 µM. n.d. not determined. n.a. not applicable. Values are means ± SEM of at least three independent experiments performed in duplicate.

**Table 2:** Protein–ligand interactions between Compound A and the opioid receptors, KOR, MOR and DOR, derived from docking experiments.

Receptor	Interaction	Functional Group	Involved Residues
KOR	Ionic interaction	Morpholine nitrogen	D138^3.32^
	Halogen bond	Chlorine	N122^2.67^
	Hydrophobic contacts	Chlorophenyl	V207^ECL2^ V118^2.63^
		2-Methylpyrrole	Y312^7.35^
		Phenyl moiety	I290^6.51^ I294^6.55^ I316^7.39^
MOR	Ionic interaction	Morpholine nitrogen	D149^3.32^
	Hydrogen bond	Morpholine oxygen	N152^3.35^
	Hydrophobic contacts	Chlorophenyl	T220^45.51/ECL2^
		2-Methylpyrrole	I324^7.39^
		Phenyl moiety	M153^3.36^ V302^6.55^ I324^7.39^
DOR	Ionic interaction + hydrogen bond	Morpholine nitrogen	D128^3.32^
	Hydrogen bond	Morpholine oxygen	N131^3.35^
	Hydrophobic contacts	Chlorophenyl	V197^ECL2^
		2-Methylpyrrol	V304^7.39^
		Phenyl moiety	Y129^3.33^ M132^3.36^ V217^5.42^ V281^6.55^

**Table 3:** Frequency of interactions between the chlorophenyl ring of Compound A and the opioid receptors.

Receptor	Interaction	Functional Group	Mean Frequency (*n* = 3)
KOR	Hydrophobic contacts	Chloride	99.1%
	Halogen bond	Chloride	13.3%
	Hydrophobic contacts	Chlorophenyl	98.4%
MOR	Hydrophobic contacts	Chloride	99.8%
	Hydrophobic contacts	Chlorophenyl	99.2%
DOR	Hydrophobic contacts	Chloride	100%
	Hydrophobic contacts	Chlorophenyl	83.2%

**Table 4:** Calculated logP and logD_7.4_ of Compound A and various small molecules KOR antagonists.

Ligand	*c*logP ^a^	*c*logD_7.4_^a^
Compound A	4.2	4.09
nor-BNI	3.13	1.55
5′-GNTI	1.72	−0.55
JDTic	3.43	1.78
JNJ-67953964	4.97	3.24
CYM-53003/BTRX-335140	3.82	2.37

^a^ Calculated using Percepta software (version 2021, ACD/Labs, Toronto, Canada) [62].

## Data Availability

Data is contained within the article and supplementary material.

## Supplementary Materials

The following supporting information can be downloaded at: https://www.mdpi.com/article/10.3390/ph15060680/s1; Figure S1: Root mean square deviation of Compound A in complex with the KOR over the simulation time; Figure S2: Root mean square deviation of the KOR backbone atoms in complex with Compound A over the simulation time; Figure S3: Root mean square deviation of Compound A in complex with the MOR over the simulation time; Figure S4: Root mean square deviation of the MOR backbone atoms in complex with Compound A over the simulation time; Figure S5: Root mean square deviation of Compound A in complex with the DOR over the simulation time; Figure S6: Root mean square deviation of the DOR backbone atoms in complex with Compound A over the simulation time. Figure S7: Root mean square fluctuation (RMSF) of KOR heavy atoms within MD simulations. Figure S8: Root mean square fluctuation (RMSF) of MOR heavy atoms within MD simulations. Figure S9: Root mean square fluctuation (RMSF) of DOR heavy atoms within MD simulations. Figure S10: Radius of gyration of the KOR in complex with Compound A over the simulation time. Figure S11: Radius of gyration of the MOR in complex with Compound A over the simulation time. Figure S12: Radius of gyration of the DOR in complex with Compound A over the simulation time. Figure S13: Solvent accessible surface area (SASA) of KOR in complex with Compound A over the simulation time. Figure S14: Solvent accessible surface area (SASA) of MOR in complex with Compound A over the simulation time. Figure S15: Solvent accessible surface area (SASA) of DOR in complex with Compound A over the simulation time. Figure S16. BOILED-Egg plot of Compound A. Figure S17. Bioavailability radar of Compound A. Table S1. Binding affinities and antagonist potencies at the KOR of compound A and know KOR antagonists. Table S2. Calculated physicochemical properties of Compound A.

## References

[1] Stein C. (2016). Opioid Receptors. Annu. Rev. Med..

[2] Hilger D., Masureel M., Kobilka B.K. (2018). Structure and dynamics of GPCR signaling complexes. Nat. Struct. Mol. Biol..

[3] Bock A., Bermudez M. (2021). Allosteric coupling and biased agonism in G protein-coupled receptors. FEBS J..

[4] Qu X., Wang D., Wu B., Jastrzebska B., Park P.S.H. (2020). Progress in GPCR structure determination. GPCRs.

[5] Sriram K., Insel P.A. (2018). G Protein-coupled receptors as targets for approved drugs: How many targets and how many drugs?. Mol. Pharmacol..

[6] Corder G., Castro D.C., Bruchas M.R., Scherrer G. (2018). Endogenous and exogenous opioids in pain. Annu. Rev. Neurosci..

[7] Darcq E., Kieffer B.L. (2018). Opioid receptors: Drivers to addiction?. Nat. Rev. Neurosci..

[8] Paul A.K., Smith C.M., Rahmatullah M., Nissapatorn V., Wilairatana P., Spetea M., Gueven N., Dietis N. (2021). Opioid analgesia and opioid-induced adverse effects: A review. Pharmaceuticals.

[9] Jacobson M.L., Browne C.A., Lucki I. (2020). Kappa opioid receptor antagonists as potential therapeutics for stress-related disorders. Annu. Rev. Pharmacol. Toxicol..

[10] Spetea M., Faheem Asim M., Wolber G., Schmidhammer H. (2013). The μ opioid receptor and ligands acting at the μ opioid receptor, as therapeutics and potential therapeutics. Curr. Pharm. Des..

[11] Pasternak G.W., Childers S.R., Pan Y.-X. (2019). Emerging insights into mu opioid pharmacology. Subst. Use Disord..

[12] Pasternak G.W., Pan Y.-X. (2013). Mu opioids and their receptors: Evolution of a concept. Pharmacol. Rev..

[13] Sobczak Ł., Goryński K. (2020). Pharmacological aspects of over-the-counter opioid drugs misuse. Molecules.

[14] Volkow N.D., Blanco C. (2021). The changing opioid crisis: Development, challenges and opportunities. Mol. Psychiatry.

[15] Liu-Chen L.-Y., Inan S. (2021). The Kappa Opioid Receptor.

[16] Lemos J.C., Chavkin C., Pasternak G. (2011). Kappa opioid receptor function. The Opiate Receptors.

[17] Cahill C., Tejeda H.A., Spetea M., Chen C., Liu-Chen L.Y. (2022). Fundamentals of the dynorphins/kappa opioid receptor system: From distribution to signaling and function. Handb. Exp. Pharmacol..

[18] Zangrandi L., Schwarzer C. (2022). The kappa opioid receptor system in temporal lobe epilepsy. Handb. Exp. Pharmacol..

[19] Spetea M., Schmidhammer H. (2022). Kappa opioid receptor ligands and pharmacology: Diphenethylamines, a class of structurally distinct, selective kappa opioid ligands. Handb. Exp. Pharmacol..

[20] Reed B., Butelman E.R., Kreek M.J. (2022). Kappa opioid receptor antagonists as potential therapeutics for mood and substance use disorders. Handb. Exp. Pharmacol..

[21] Browne C.A., Wulf H., Lucki I. (2022). Kappa Opioid receptors in the pathology and treatment of major depressive disorder. Handb. Exp. Pharmacol..

[22] Schmidhammer H., Erli F., Guerrieri E., Spetea M. (2020). Development of diphenethylamines as selective kappa opioid receptor ligands and their pharmacological activities. Molecules.

[23] Aldrich J.V., McLaughlin J.P. (2022). Peptide Kappa opioid receptor ligands and their potential for drug development. Handb. Exp. Pharmacol..

[24] Prisinzano T.E. (2013). Neoclerodanes as Atypical opioid receptor ligands: 2012 David W. Robertson award for excellence in medicinal chemistry. J. Med. Chem..

[25] Nagase H., Hayakawa J., Kawamura K., Kawai K., Takezawa Y., Matsuura H., Tajima C., Endo T. (1998). Discovery of a structurally novel opioid k-agonist derived from 4, 5-epoxymorphinan. Chem. Pharm. Bull..

[26] Vanderah T.W., Largent-Milnes T., Lai J., Porreca F., Houghten R.A., Menzaghi F., Wisniewski K., Stalewski J., Sueiras-Diaz J., Galyean R. (2008). Novel D-amino acid tetrapeptides produce potent antinociception by selectively acting at peripheral κ-opioid receptors. Eur. J. Pharmacol..

[27] Deeks E.D. (2021). Difelikefalin: First approval. Drugs.

[28] Inan S., Cowan A. (2022). Antipruritic effects of kappa opioid receptor agonists: Evidence from rodents to humans. Handb. Exp. Pharmacol..

[29] Miyamoto Y., Oh T., Aihara E., Ando A. (2022). Clinical profiles of nalfurafine hydrochloride for the treatment of pruritus patients. Handb. Exp. Pharmacol..

[30] Albert-Vartanian A., Boyd M., Hall A., Morgado S., Nguyen E., Nguyen V., Patel S., Russo L., Shao A., Raffa R. (2016). Will peripherally restricted kappa-opioid receptor agonists (pKORA s) relieve pain with less opioid adverse effects and abuse potential?. J. Clin. Pharm. Ther..

[31] Lazenka M.F. (2022). Antinociceptive Effects of kappa-opioid receptor agonists. Handb. Exp. Pharmacol..

[32] Paton K.F., Atigari D.V., Kaska S., Prisinzano T., Kivell B.M. (2020). Strategies for Developing κ opioid receptor agonists for the treatment of pain with fewer side effects. J. Pharmacol. Exp. Ther..

[33] Kaski S.W., White A.N., Gross J.D., Siderovski D.P. (2021). Potential for kappa-opioid receptor agonists to engineer nonaddictive analgesics: A narrative review. Anesth. Analg..

[34] Carroll F.I., Carlezon W.A. (2013). Development of κ opioid receptor antagonists. J. Med. Chem..

[35] Portoghese P.S., Lipkowski A., Takemori A. (1987). Binaltorphimine and nor-binaltorphimine, potent and selective k-opioid receptor antagonists. Life Sci..

[36] Jones R.M., Portoghese P.S. (2000). 5′-Guanidinonaltrindole, a highly selective and potent κ-opioid receptor antagonist. Eur. J. Pharmacol..

[37] Thomas J.B., Atkinson R.N., Rothman R.B., Fix S.E., Mascarella S.W., Vinson N.A., Xu H., Dersch C.M., Lu Y.-F., Cantrell B.E. (2001). Identification of the first trans-(3 R, 4 R)-dimethyl-4-(3-hydroxyphenyl) piperidine derivative to possess highly potent and selective opioid κ receptor antagonist activity. J. Med. Chem..

[38] Buda J.J., Carroll F.I., Kosten T.R., Swearingen D., Walters B.B. (2015). A double-blind, placebo-controlled trial to evaluate the safety, tolerability, and pharmacokinetics of single, escalating oral doses of JDTic. Neuropsychopharmacology.

[39] Munro T.A., Berry L.M., Van’t Veer A., Béguin C., Carroll F., Zhao Z., Carlezon W.A., Cohen B.M. (2012). Long-acting κ opioid antagonists nor-BNI, GNTI and JDTic: Pharmacokinetics in mice and lipophilicity. BMC Pharmacol..

[40] Black S.L., Chauvignac C., Grundt P., Miller C.N., Wood S., Traynor J.R., Lewis J.W., Husbands S.M. (2003). Guanidino N-substituted and N,N-disubstituted derivatives of the kappa-opioid antagonist GNTI. J. Med. Chem..

[41] Endoh T., Matsuura H., Tanaka C., Nagase H. (1992). Nor-binaltorphimine: A potent and selective kappa-opioid receptor antagonist with long-lasting activity in vivo. Arch. Int. Pharmacodyn. Ther..

[42] Rorick-Kehn L.M., Witkin J.M., Statnick M.A., Eberle E.L., McKinzie J.H., Kahl S.D., Forster B.M., Wong C.J., Li X., Crile R.S. (2014). LY2456302 is a novel, potent, orally-bioavailable small molecule kappa-selective antagonist with activity in animal models predictive of efficacy in mood and addictive disorders. Neuropharmacology.

[43] Guerrero M., Urbano M., Kim E.-K., Gamo A.M., Riley S., Abgaryan L., Leaf N., Van Orden L.J., Brown S.J., Xie J.Y. (2019). Design and synthesis of a novel and selective kappa opioid receptor (KOR) antagonist (BTRX-335140). J. Med. Chem..

[44] Patkar K.A., Yan X., Murray T.F., Aldrich J.V. (2005). [N^α^-BenzylTyr ^1^,cyclo(d-Asp^5^,Dap^8^)]-dynorphin A-(1−11)NH_2_ cyclized in the “Address” Domain is a novel κ-Opioid receptor antagonist. J. Med. Chem..

[45] Joshi A.A., Murray T.F., Aldrich J.V. (2015). Structure-Activity relationships of the peptide kappa opioid receptor antagonist zyklophin. J. Med. Chem..

[46] Wu H., Wacker D., Mileni M., Katritch V., Han G.W., Vardy E., Liu W., Thompson A.A., Huang X.-P., Carroll F. (2012). Structure of the human κ-opioid receptor in complex with JDTic. Nature.

[47] Che T., Majumdar S., Zaidi S.A., Ondachi P., McCorvy J.D., Wang S., Mosier P.D., Uprety R., Vardy E., Krumm B.E. (2018). Structure of the nanobody-stabilized active state of the kappa opioid receptor. Cell.

[48] Che T., English J., Krumm B.E., Kim K., Pardon E., Olsen R.H.J., Wang S., Zhang S., Diberto J.F., Sciaky N. (2020). Nanobody-enabled monitoring of kappa opioid receptor states. Nat. Commun..

[49] Zaidi S.A., Katritch V. (2022). Structural Characterization of KOR Inactive and active states for 3D pharmacology and drug discovery. Handb. Exp. Pharmacol..

[50] Kaserer T., Lantero A., Schmidhammer H., Spetea M., Schuster D. (2016). μ Opioid receptor: Novel antagonists and structural modeling. Sci. Rep..

[51] Erli F., Guerrieri E., Ben Haddou T., Lantero A., Mairegger M., Schmidhammer H., Spetea M. (2017). Highly Potent and selective new diphenethylamines interacting with the κ-opioid receptor: Synthesis, pharmacology, and structure-activity relationships. J. Med. Chem..

[52] Dumitrascuta M., Bermudez M., Trovato O., De Neve J., Ballet S., Wolber G., Spetea M. (2021). Antinociceptive efficacy of the µ-opioid/nociceptin peptide-based hybrid KGNOP1 in inflammatory pain without rewarding effects in mice: An experimental assessment and molecular docking. Molecules.

[53] Manglik A., Kruse A.C., Kobilka T.S., Thian F.S., Mathiesen J.M., Sunahara R.K., Pardo L., Weis W.I., Kobilka B.K., Granier S. (2012). Crystal structure of the µ-opioid receptor bound to a morphinan antagonist. Nature.

[54] Fenalti G., Giguere P.M., Katritch V., Huang X.-P., Thompson A.A., Cherezov V., Roth B.L., Stevens R.C. (2014). Molecular control of δ-opioid receptor signalling. Nature.

[55] Ballesteros J.A., Weinstein H., Sealfon S.C. (1995). [19] Integrated methods for the construction of three-dimensional models and computational probing of structure-function relations in G protein-coupled receptors. Methods in Neurosciences.

[56] Fenalti G., Zatsepin N.A., Betti C., Giguere P., Han G.W., Ishchenko A., Liu W., Guillemyn K., Zhang H., James D. (2015). Structural basis for bifunctional peptide recognition at human δ-opioid receptor. Nat. Struct. Mol. Biol..

[57] Vo Q.N., Mahinthichaichan P., Shen J., Ellis C.R. (2021). How μ-opioid receptor recognizes fentanyl. Nat. Commun..

[58] Sydow D. (2015). Dynophores: Novel Dynamic Pharmacophores Implementation of Pharmacophore Generation Based on Molecular Dynamics Trajectories and Their Graphical Representation.

[59] Avdeef A., Testa B. (2002). Physicochemical profiling in drug research: A brief survey of the state-of-the-art of experimental techniques. Cell. Mol. Life Sci..

[60] Faller B., Rekka E.A., Kourounakis P.N. (2008). Physicochemical profiling in early drug discovery: New challenges at the age of high-throughput screen and combinatorial chemistry. Chemistry and Molecular Aspects of Drug Design and Action.

[61] Habgood M., Begley D., Abbott N. (2000). Determinants of passive drug entry into the central nervous system. Cell. Mol. Neurobiol..

[62] (2021). ACD/Percepta.

[63] Daina A., Zoete V. (2016). A boiled-egg to predict gastrointestinal absorption and brain penetration of small molecules. ChemMedChem.

[64] Daina A., Michielin O., Zoete V. (2017). SwissADME: A free web tool to evaluate pharmacokinetics, drug-likeness and medicinal chemistry friendliness of small molecules. Sci. Rep..

[65] Bradford M.M. (1976). A rapid and sensitive method for the quantitation of microgram quantities of protein utilizing the principle of protein-dye binding. Anal. Biochem..

[66] Cheng Y.-C., Prusoff W.H. (1973). Relationship between the inhibition constant (KI) and the concentration of inhibitor which causes 50 per cent inhibition (I50) of an enzymatic reaction. Biochem. Pharmacol..

[67] Berman H., Henrick K., Nakamura H. (2003). Announcing the worldwide Protein Data Bank. Nat. Struct. Biol..

[68] Molecular Operating Environment (MOE), C.C.G.U., Sherbooke St. West, Suite #910, Montreal, QC, Canada, H3A 2R7, 2021. https://www.chemcomp.com/Products.htm.

[69] The UniProt Consortium (2021). UniProt: The universal protein knowledgebase in 2021. Nucleic Acids Res..

[70] Ramachandran G.N., Ramakrishnan C., Sasisekharan V. (1963). Stereochemistry of polypeptide chain configurations. J. Mol. Biol..

[71] Zhu S. (2019). Validation of the Generalized Force Fields GAFF, CGenFF, OPLS-AA, and PRODRGFF by Testing Against Experimental Osmotic Coefficient Data for Small Drug-Like Molecules. J. Chem. Inf. Model..

[72] Labute P. (2008). The generalized Born/volume integral implicit solvent model: Estimation of the free energy of hydration using London dispersion instead of atomic surface area. J. Comput. Chem..

[73] Labute P. (2009). Protonate3D: Assignment of ionization states and hydrogen coordinates to macromolecular structures. Proteins.

[74] Vardy E., Mosier P.D., Frankowski K.J., Wu H., Katritch V., Westkaemper R.B., Aubé J., Stevens R.C., Roth B.L. (2013). Chemotype-selective modes of action of κ-opioid receptor agonists. J. Biol. Chem..

[75] Chavkin C., McLaughlin J.P., Celver J.P. (2001). Regulation of opioid receptor function by chronic agonist exposure: Constitutive activity and desensitization. Mol. Pharmacol..

[76] Claff T., Yu J., Blais V., Patel N., Martin C., Wu L., Han G.W., Holleran B.J., van der Poorten O., White K.L. (2019). Elucidating the active δ-opioid receptor crystal structure with peptide and small-molecule agonists. Sci. Adv..

[77] Befort K., Zilliox C., Filliol D., Yue S., Kieffer B.L. (1999). Constitutive activation of the delta opioid receptor by mutations in transmembrane domains III and VII. J. Biol. Chem..

[78] Décaillot F.M., Befort K., Filliol D., Yue S., Walker P., Kieffer B.L. (2003). Opioid receptor random mutagenesis reveals a mechanism for G protein-coupled receptor activation. Nat. Struct. Biol..

[79] 3D Structure Generator CORINA Classic Molecular Networks GmbH, Nuremberg, Germany. https://mn-am.com/products/corina/.

[80] Gasteiger J., Rudolph C., Sadowski J. (1990). Automatic generation of 3D-atomic coordinates for organic molecules. Tetrahedron Comput. Methodol..

[81] Jones G., Willett P., Glen R.C., Leach A.R., Taylor R. (1997). Development and validation of a genetic algorithm for flexible docking. J. Mol. Biol..

[82] Evers A., Hessler G., Matter H., Klabunde T. (2005). Virtual screening of biogenic amine-binding G-protein coupled receptors: Comparative evaluation of protein- and ligand-based virtual screening protocols. J. Med. Chem..

[83] Verdonk M.L., Cole J.C., Hartshorn M.J., Murray C.W., Taylor R.D. (2003). Improved protein-ligand docking using GOLD. Proteins.

[84] Halgren T.A. (1996). Merck molecular force field. I. Basis, form, scope, parameterization, and performance of MMFF94. J. Comput. Chem..

[85] Halgren T.A. (1996). Merck molecular force field. II. MMFF94 van der Waals and electrostatic parameters for intermolecular interactions. J. Comput. Chem..

[86] Halgren T.A. (1996). Merck molecular force field. III. Molecular geometries and vibrational frequencies for MMFF94. J. Comput. Chem..

[87] Halgren T.A., Nachbar R.B. (1996). Merck molecular force field. IV. Conformational energies and geometries for MMFF94. J. Comput. Chem..

[88] Halgren T.A. (1996). Merck molecular force field. V. Extension of MMFF94 using experimental data, additional computational data, and empirical rules. J. Comput. Chem..

[89] Wolber G., Dornhofer A.A., Langer T. (2006). Efficient overlay of small organic molecules using 3D pharmacophores. J. Comput. Aided Mol. Des..

[90] Wolber G., Langer T. (2005). LigandScout: 3-D pharmacophores derived from protein-bound ligands and their use as virtual screening filters. J. Chem. Inf. Model..

[91] (2020). Schrödinger Release-4: Maestro.

[92] Jorgensen W.L., Schyman P. (2012). Treatment of Halogen Bonding in the OPLS-AA Force Field: Application to Potent Anti-HIV Agents. J. Chem. Theory Comput..

[93] Ponder J.W., Case D.A. (2003). Force Fields for protein simulations. Advances in Protein Chemistry.

[94] Bowers K.J., Chow D.E., Xu H., Dror R.O., Eastwood M.P., Gregersen B.A., Klepeis J.L., Kolossvary I., Moraes M.A., Sacerdoti F.D. Scalable Algorithms for Molecular Dynamics Simulations on Commodity Clusters. Proceedings of the ACM/IEEE Conference on Supercomputing (SC06).

[95] Lomize M.A., Pogozheva I.D., Joo H., Mosberg H.I., Lomize A.L. (2012). OPM database and PPM web server: Resources for positioning of proteins in membranes. Nucleic Acids Res..

[96] Jorgensen W.L., Chandrasekhar J., Madura J.D., Impey R.W., Klein M.L. (1983). Comparison of simple potential functions for simulating liquid water. J. Chem. Phys..

[97] Bock A., Bermudez M., Krebs F., Matera C., Chirinda B., Sydow D., Dallanoce C., Holzgrabe U., Amici M.d., Lohse M.J. (2016). Ligand binding ensembles determine graded agonist efficacies at a G protein-coupled receptor. J. Biol. Chem..

[98] Humphrey W., Dalke A., Schulten K. (1996). VMD: Visual molecular dynamics. J. Mol. Graph..

