# Supplementary material for: In Vitro, In Vivo and In Silico Characterization of a Novel Kappa-Opioid Receptor Antagonist
Source: Pharmaceuticals (Basel). 2022 May 28;15(6):680. doi: 10.3390/ph15060680 (PMC9229160; doi:10.3390/ph15060680)
Supplement: Supplementary file 1 [file pharmaceuticals-15-00680-s001.zip › pharmaceuticals-1724236-supplementary.pdf]

Supporting Materials

In vitro, in vivo and *in silico* characterization of a novel kappa-opioid receptor antagonist

Kristina Puls[1], Aina-Leonor Olivé-Marti[2], Szymon Pach[1], Birgit Pinter[2], Filippo Erli[2], Gerhard Wolber[1*], Mariana Spetea[2*]

[1]Department of Pharmaceutical Chemistry, Institute of Pharmacy, Freie Universität Berlin; Königin-Luise-Str. 2+4, D-14195 Berlin, Germany; kristina.puls@fu-berlin.de (K.P.), s.pach@fu-berlin.de (S.P.), gerhard.wolber@fu-berlin.de (G.W.)
[2]Department of Pharmaceutical Chemistry, Institute of Pharmacy and Center for Molecular Biosciences Innsbruck (CMBI), University of Innsbruck, Innrain 80-82, 6020 Innsbruck, Austria; aina-leonor.olive-marti@uibk.ac.at (A-L.O-M.), birgit-pinter@gmax.at (B.P.), f.erli@hotmail.it (F.E.), mariana.spetea@uibk.ac.at (M.S.)

*Correspondence: gerhard.wolber@fu-berlin.de (G.W.), Tel.: +49-30-838-52686; mariana.spetea@uibk.ac.at (M.S.), Tel.: +43-512-507-58277

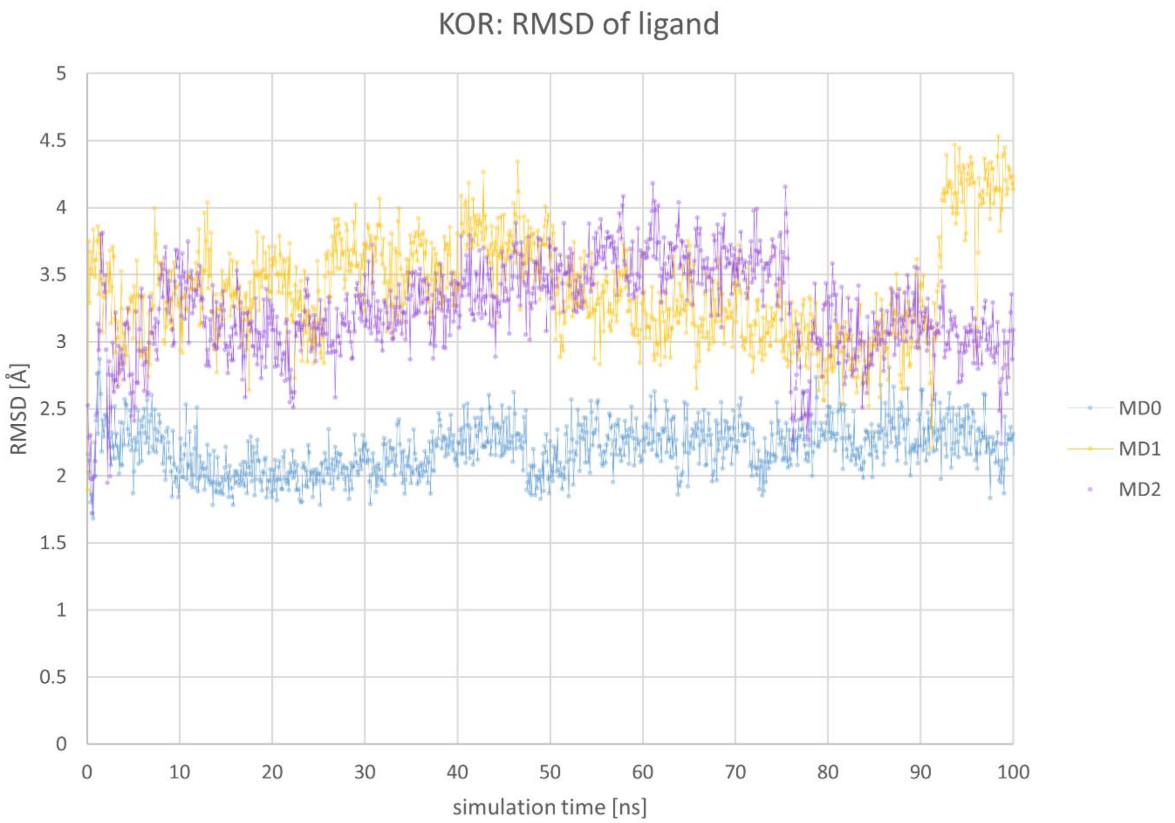

Figure S1. Root mean square deviation of Compound A in complex with the KOR over the simulation time.

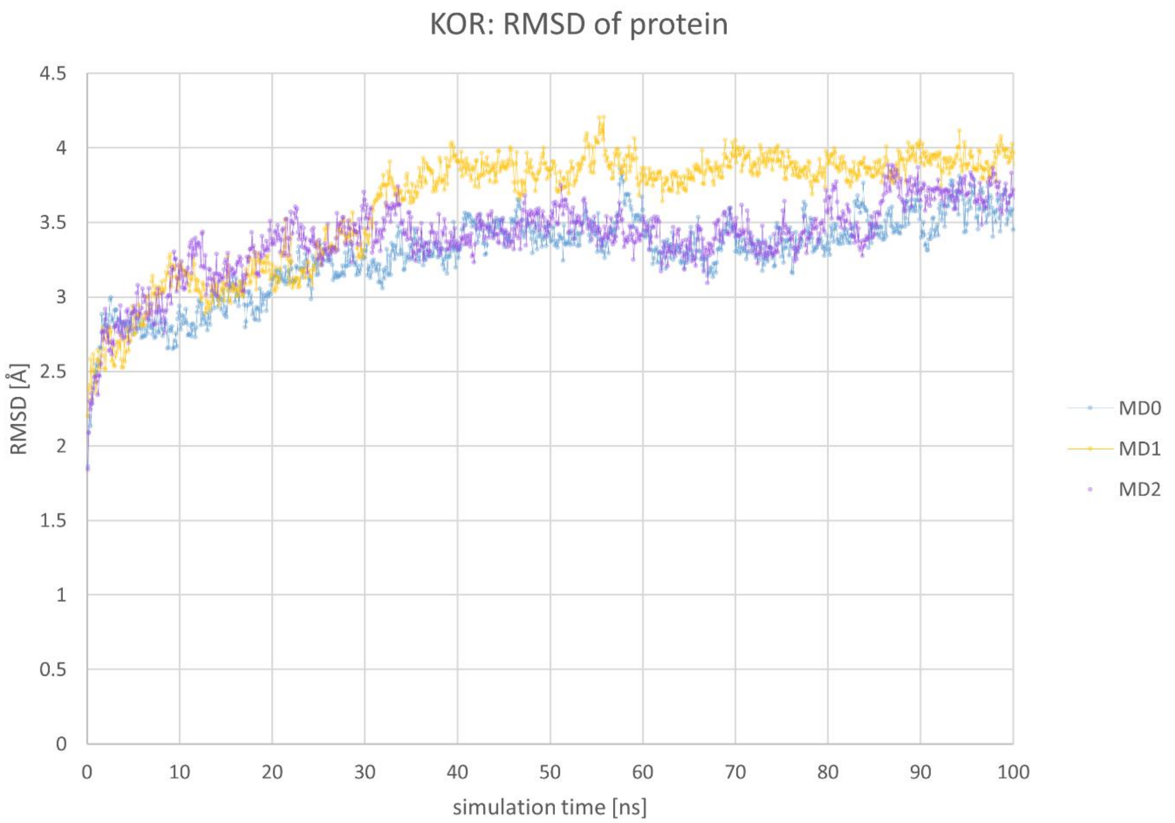

Figure S2. Root mean square deviation of the KOR backbone atoms in complex with Compound A over the simulation time.

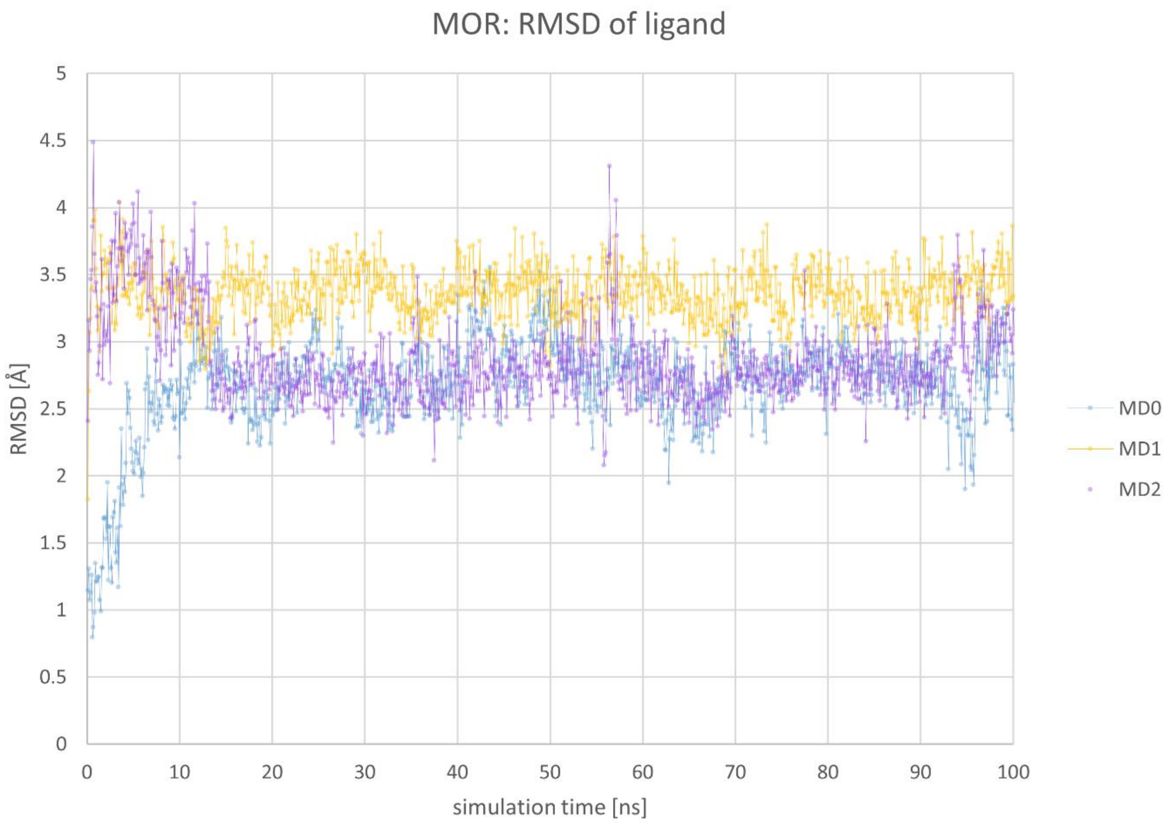

Figure S3. Root mean square deviation of Compound A in complex with the MOR over the simulation time.

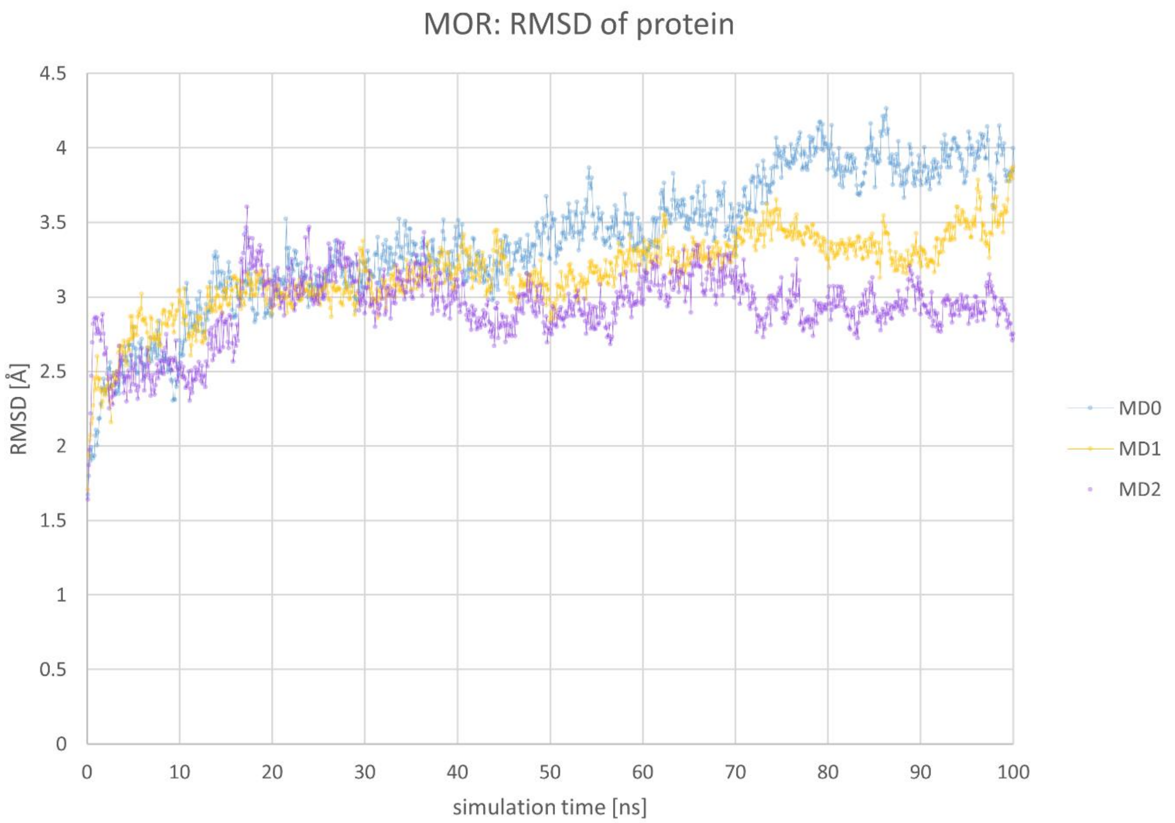

Figure S4. Root mean square deviation of the MOR backbone atoms in complex with Compound A over the simulation time.

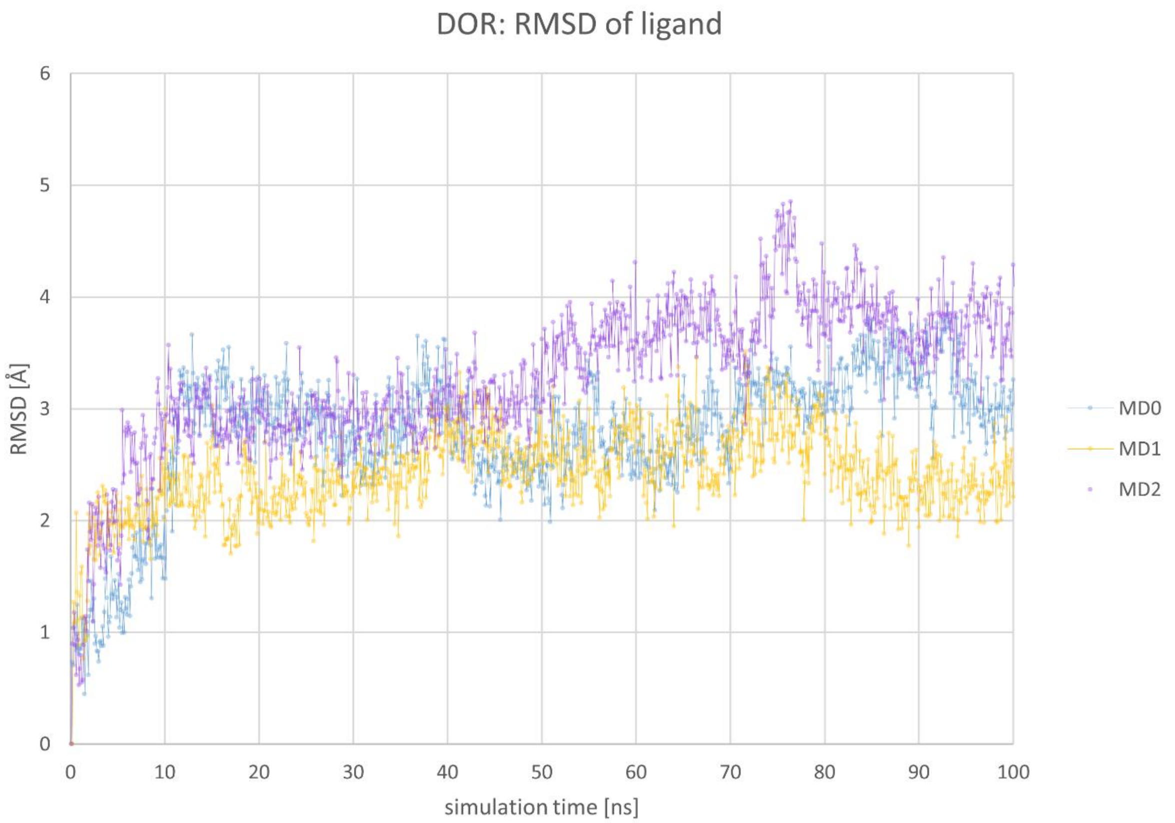

Figure S5. Root mean square deviation of Compound A in complex with the DOR over the simulation time.

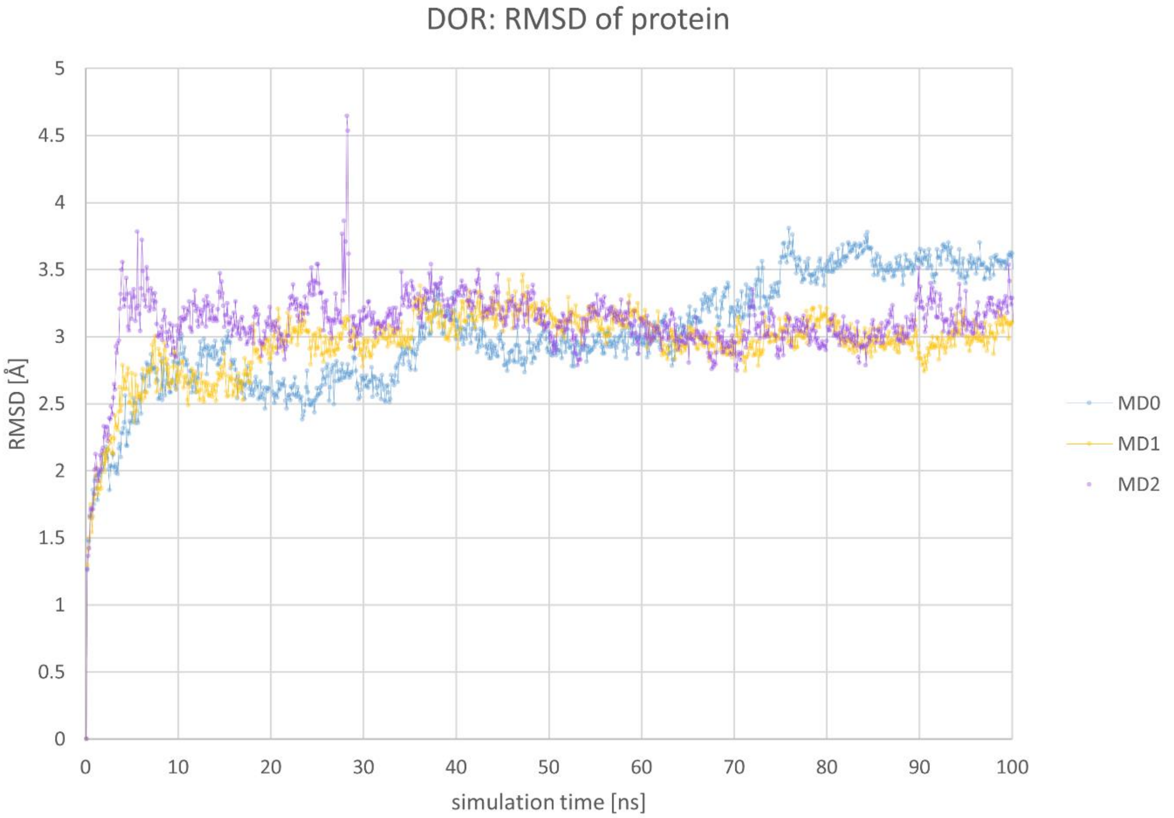

Figure S6. Root mean square deviation of the DOR backbone atoms in complex with Compound A over the simulation time.

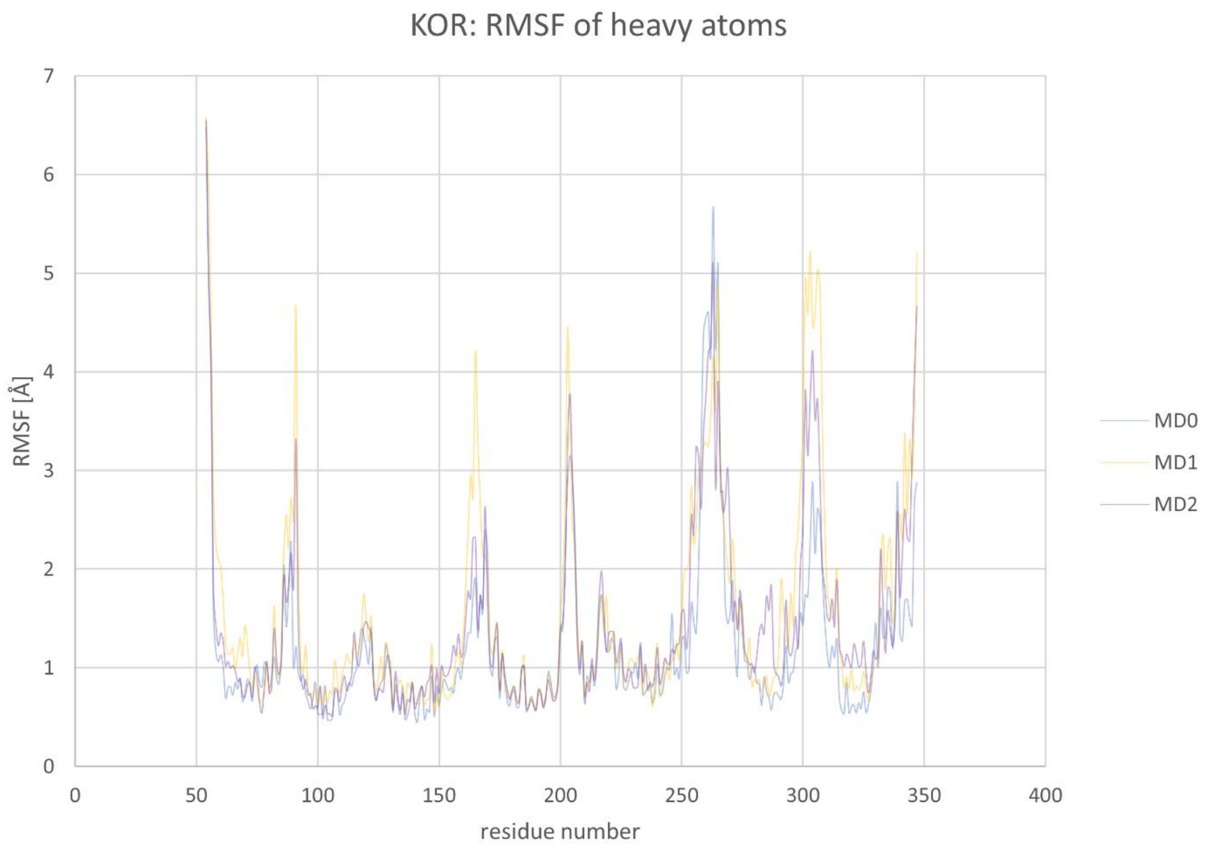

Figure S7. Root mean square fluctuation (RMSF) of KOR heavy atoms within MD simulations.

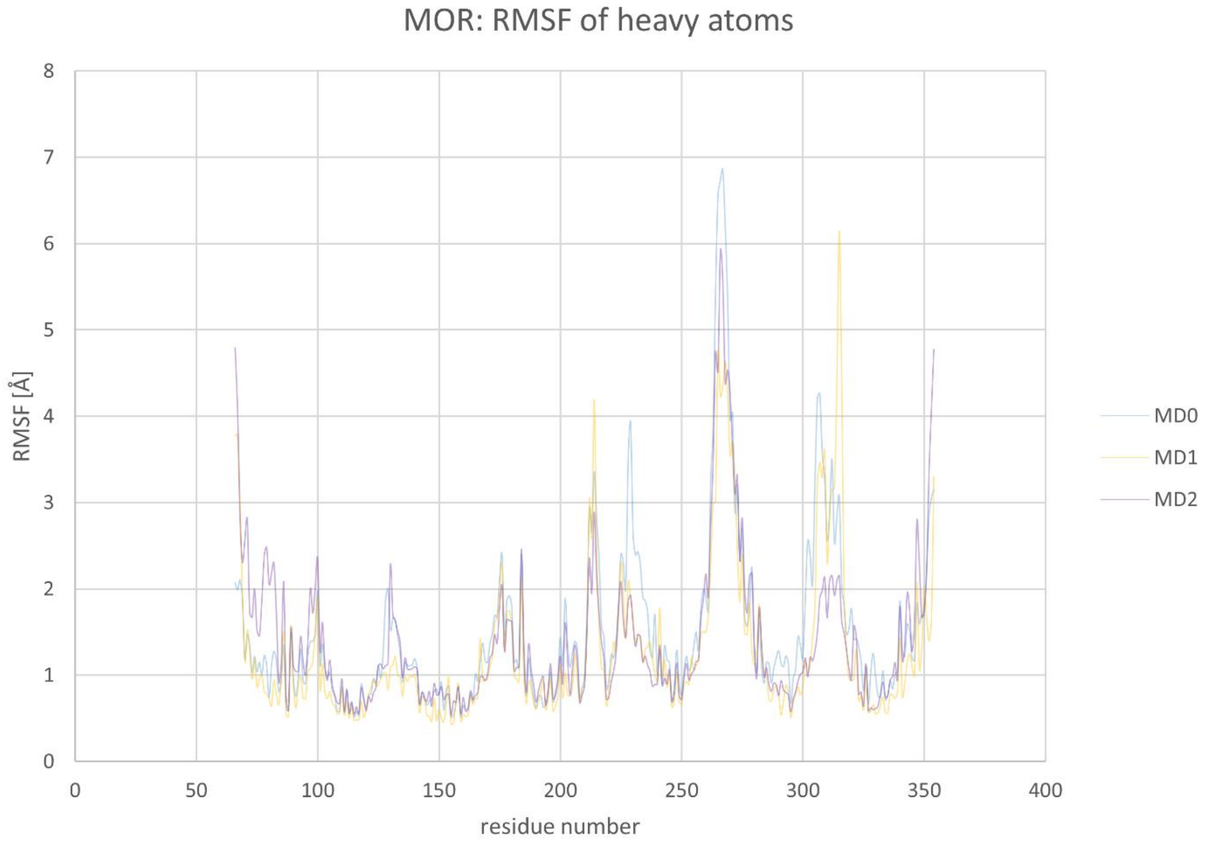

Figure S8. Root mean square fluctuation (RMSF) of MOR heavy atoms within MD simulations.

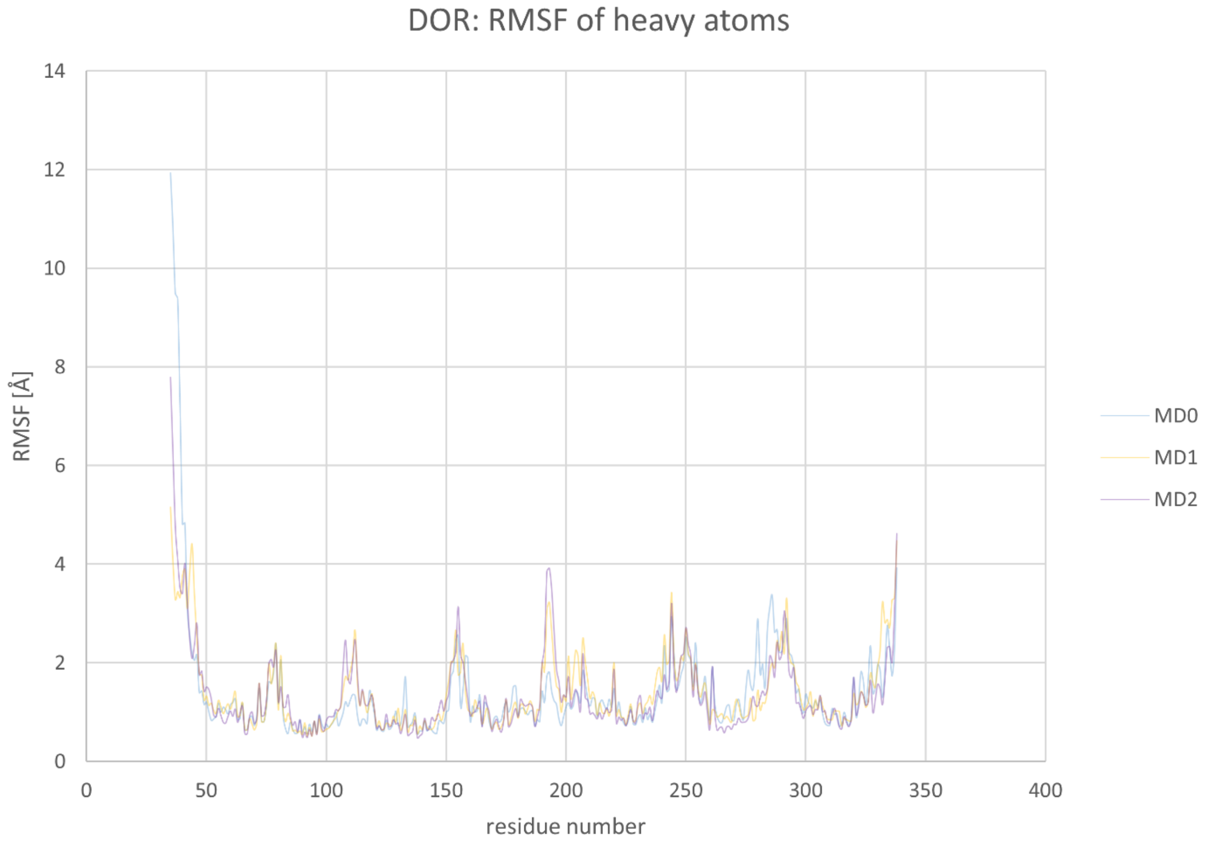

Figure S9. Root mean square fluctuation (RMSF) of DOR heavy atoms within MD simulations.

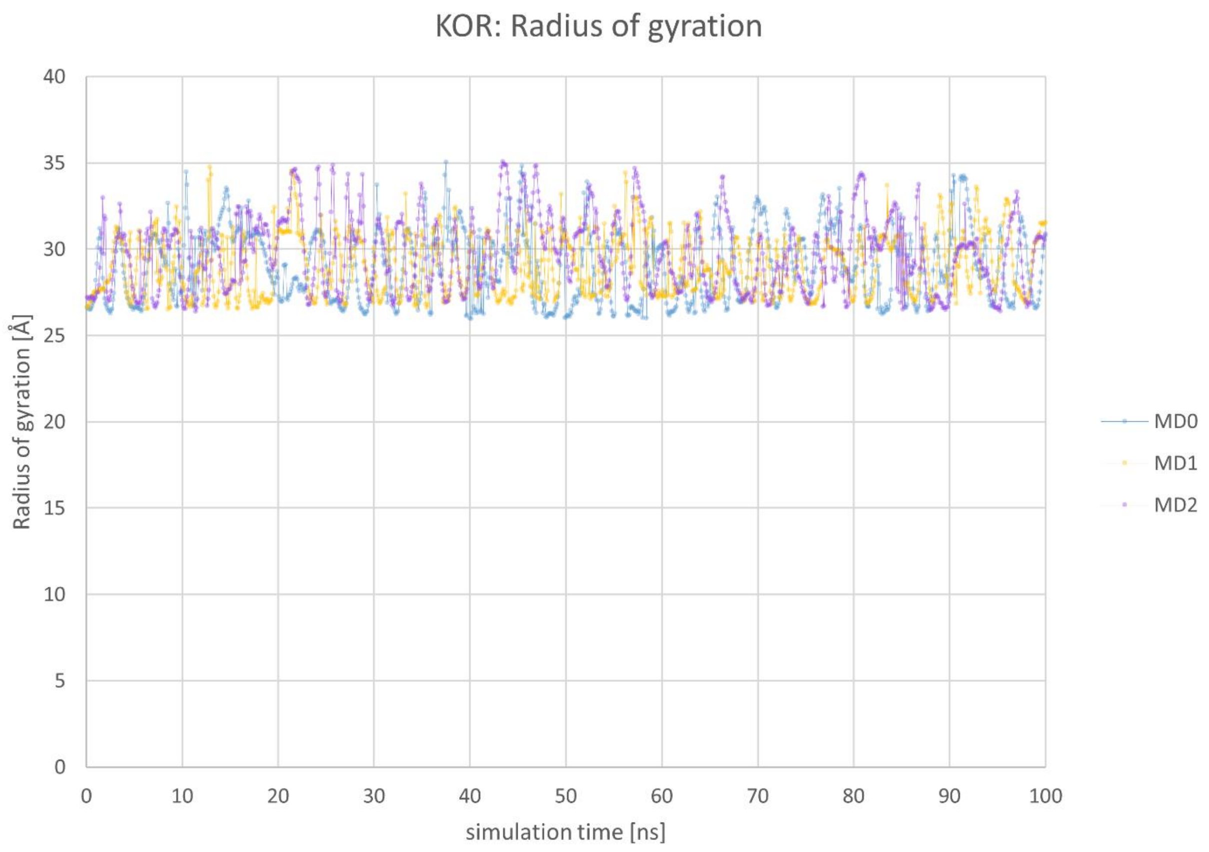

Figure S10. Radius of gyration of the KOR in complex with Compound A over the simulation time. The radius of gyration values remain stable over the simulation time corresponding to a similar KOR compactness along the MD simulations.

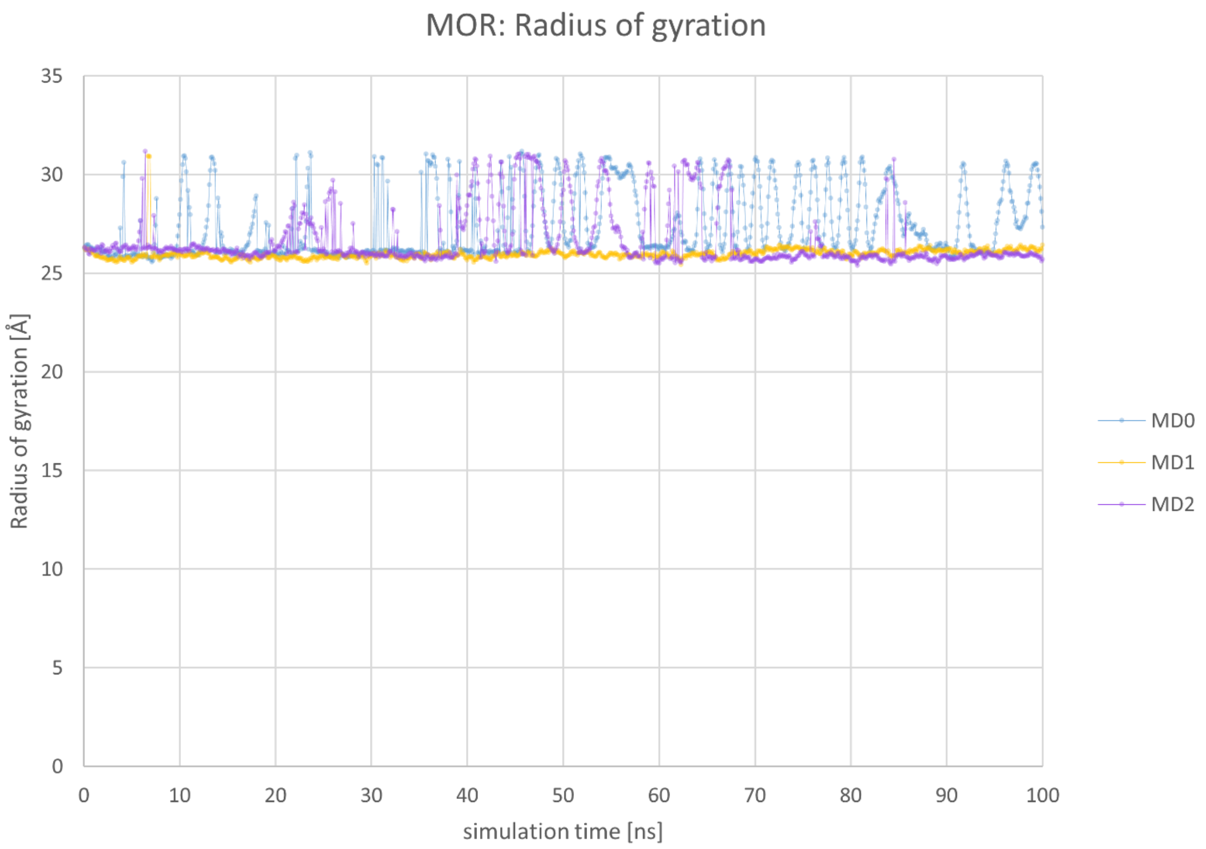

Figure S11. Radius of gyration of the MOR in complex with Compound A over the simulation time. The radius of gyration values remain stable over the simulation time corresponding to a similar MOR compactness along the MD simulations.

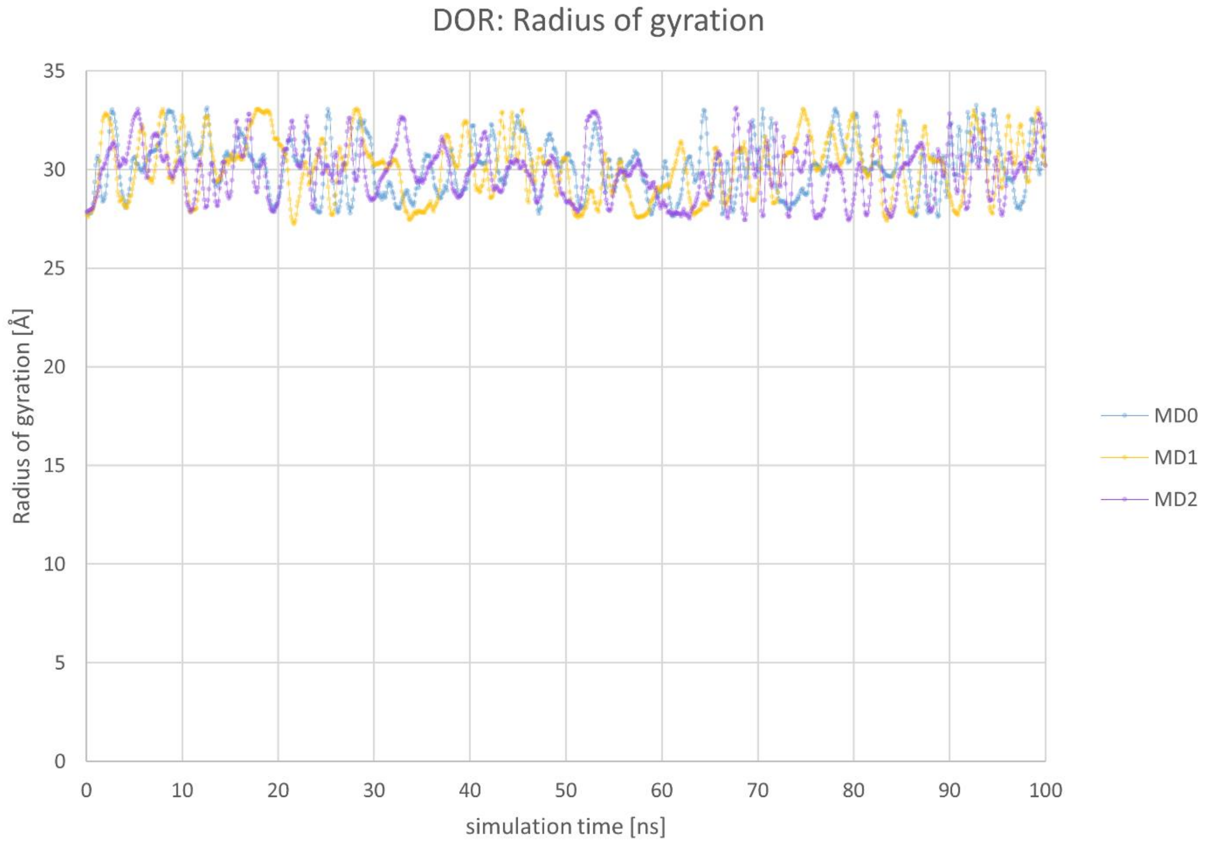

Figure S12. Radius of gyration of the DOR in complex with Compound A over the simulation time. The radius of gyration values remain stable over the simulation time corresponding to a similar DOR compactness along the MD simulations.

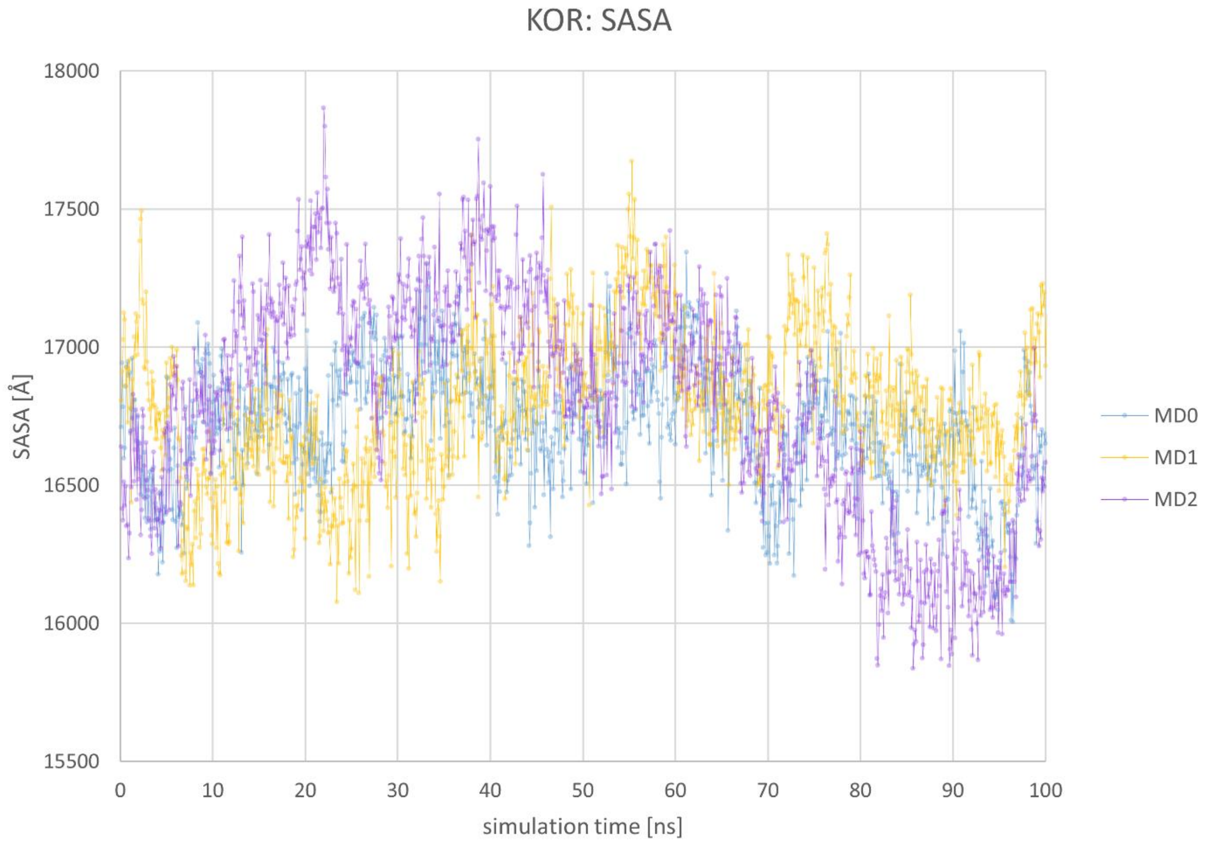

Figure S13. Solvent accessible surface area (SASA) of KOR in complex with Compound A over the simulation time. No strong increase in SASA was observed correlating with stable receptor folding.

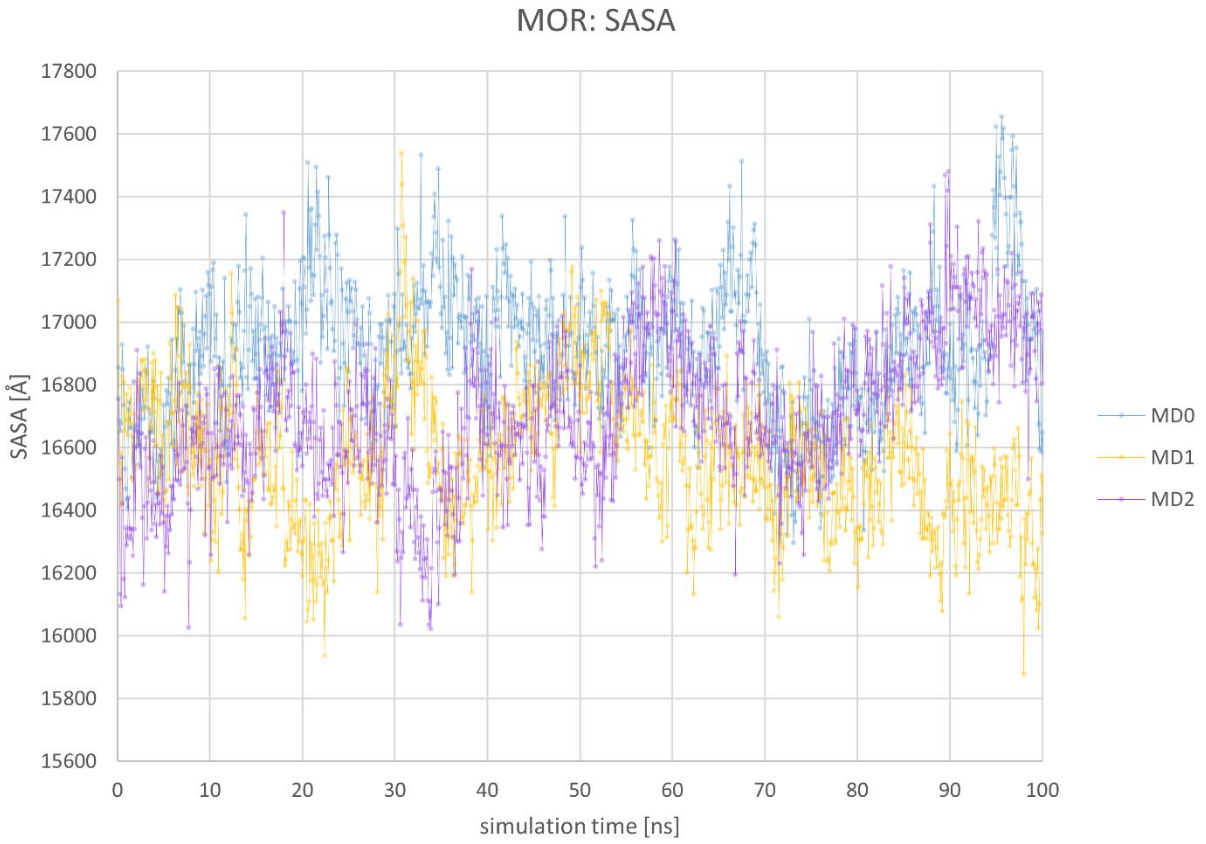

Figure S14. Solvent accessible surface area (SASA) of MOR in complex with Compound A over the simulation time. No strong increase in SASA was observed correlating with stable receptor folding.

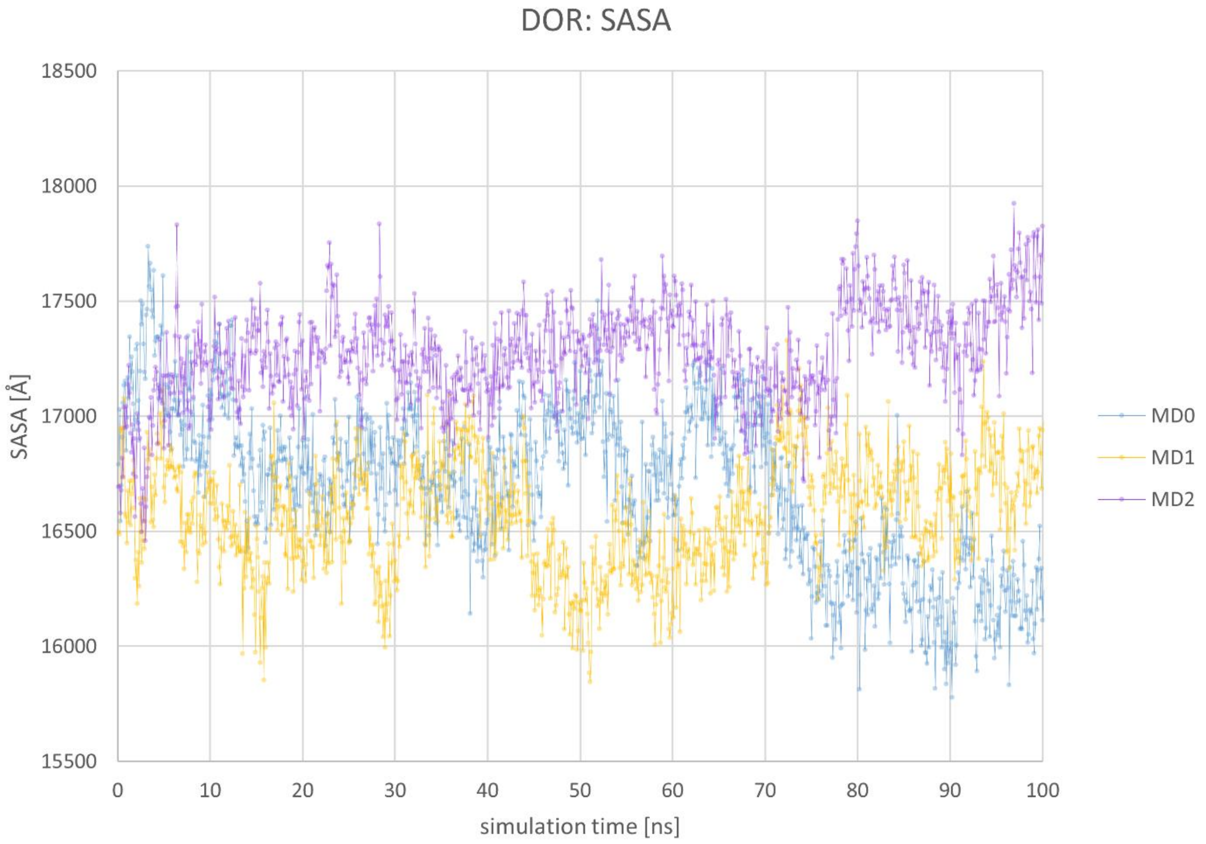

Figure S15. Solvent accessible surface area (SASA) of DOR in complex with Compound A over the simulation time. No strong increase in SASA was observed correlating with stable receptor folding.

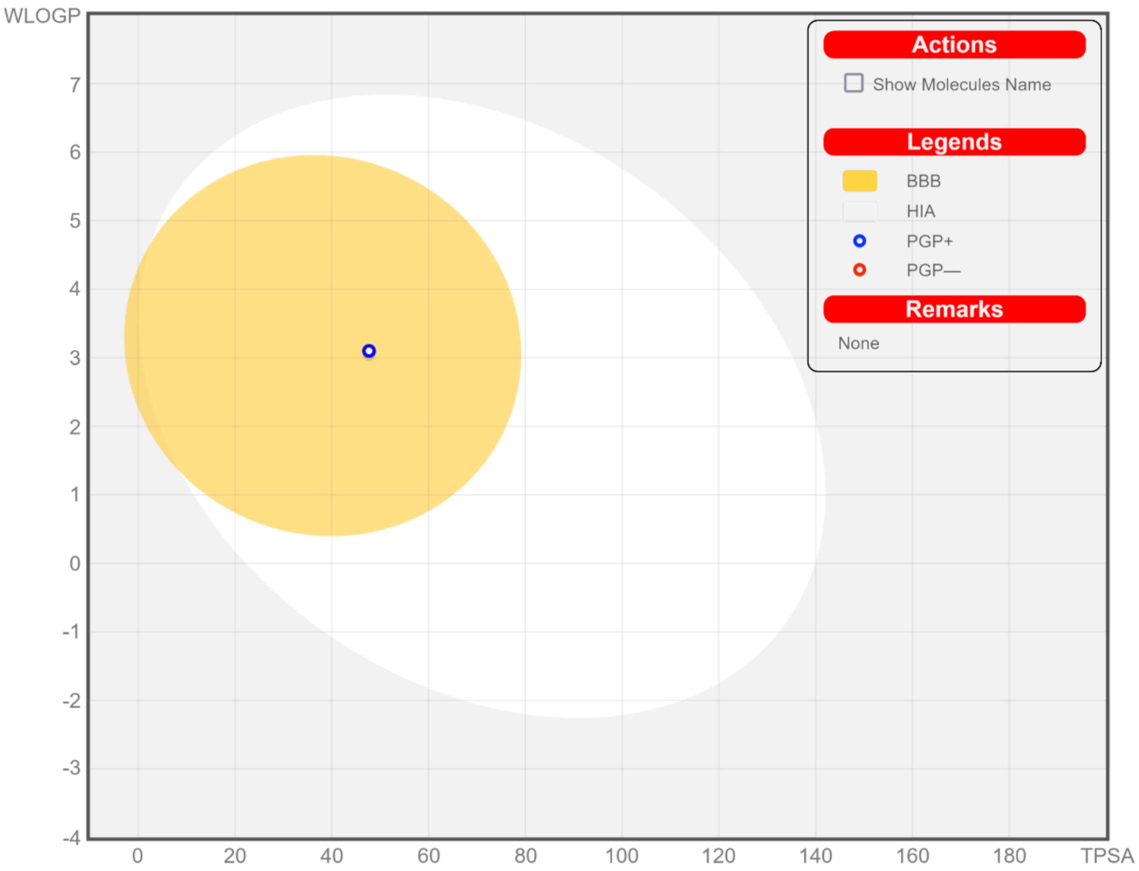

Figure S16. BOILED-Egg plot of Compound A calculated using SwissADME web tool [1]. The BOILED-Egg method (Brain or IntestinaL EstimateD permeation method) calculated lipophilicity (WLOGP, a logP method developed by Wildman and Crippen) and polarity (topological polar surface area, TPSA) to estimate the permeation capabilities of small molecules [2]. Compound A was predicted to have high gastrointestinal absorption and to readily pass the brain- barrier barrier, but also as a permeability-glycoprotein substrate. These findings are in accordance with the previous hypothesis of Compound A being a CNS penetrant. Abbreviations: HIA: passive human gastrointestinal absorption, PGP: Permeability glycoprotein.

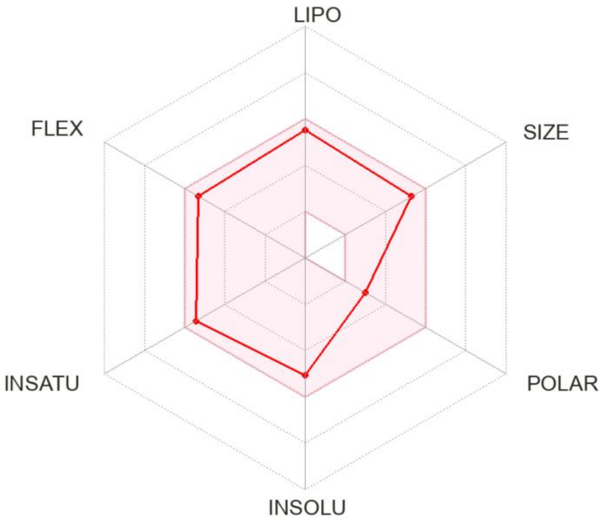

Figure S17. Bioavailability radar of Compound A. The bioavailability radar includes information about Compound A's lipophilicity (LIPO), molecular weight (SIZE), polarity (POLAR), solubility (INSOLU), flexibility (FLEX), and saturation (INSATU), all calculated using the SwissADME web tool [1]. Compound A is predicted orally available as all calculated physicochemical properties are within the optimal range (LIPO: 0.7 < XLOGP3 < +5.0; SIZE: 150 g/mol < MV < 500 g/mol; POLAR: 20Å^2 < TPSA < 130 Å^2; INSOLU: -6 < Log S (ESOL) < 0; INSATU: 0.25 < Fraction Csp3 < 1; FLEX: 0 < No. of rotatable bonds < 9) indicated in pink.

Table S1. Binding affinities and antagonist potencies at the KOR of Compound A and known KOR antagonists

Ligand	Binding affinity[a] K_i	Antagonist activity[b] K_e	Reference
Compound A	1.53 μM	1.53 μM	
nor-BNI	0.153 nM	0.798 nM	[3]
5′-GNTI	0.18 nM	0.04 nM	[4]
JDTic	0.031 nM	0.098 nM	[3]
JNJ-67953964/LY2456302	0.807 nM	0.813 nM	[3]
CYM-53003/BTRX-335140	-[c]	0.8 nM[d]	[5]
Zyklophin	95.2 nM	336 nM	[6]

[a]Determined in the radioligand binding assay using recombinant cells expressing the human KOR; [b]Determined in the [^{35}S]GTPγS binding assay using recombinant cells expressing the human KOR. [c]- Not reported. [d]Reported as IC_{50} value.

Table S2. Calculated physicochemical properties of Compound A[a] to predict its bioavailability

Physicochemical property	Parameter	Predicted value[b]
Lipophilicity (LIPO)	Log $P_{o/w}$ (XLOGP3)	4.14
Molecular weight (SIZE)	Molecular weight	438.97 g/mol
Polarity (POLAR)	TPSA	47.70 Å^2
Solubility (INSOLU)	Log S (ESOL)	-5.05
Flexibility (FLEX)	Fraction Csp3	0.32
Saturation (INSATU)	Number of rotatable bonds	8

[a]For Compound A in the protonated state. [b]Calculated using SwissADME web tool [1]. Abbreviations: TPSA: topological polar surface area, ESOL: estimated solubility, log S: decimal logarithm of the molar solubility in water, Csp3: total carbon count of the molecule.

References

1. Daina, A.; Michielin, O.; Zoete, V. SwissADME: a free web tool to evaluate pharmacokinetics, drug-likeness and medicinal chemistry friendliness of small molecules. *Sci. Rep.* **2017**, *7*, 42717.
2. Daina, A.; Zoete, V. A boiled-egg to predict gastrointestinal absorption and brain penetration of small molecules. *ChemMedChem* **2016**, *11*, 1117-1121.
3. Rorick-Kehn, L. M.; Witkin, J. M.; Statnick, M. A.; Eberle, E. L.; McKinzie, J. H.; Kahl, S. D.; Forster, B. M.; Wong, C. J.; Li, X.; Crile, R. S.; Shaw, D. B.; Sahr, A. E.; Adams, B. L.; Quimby, S. J.; Diaz, N.; Jimenez, A.; Pedregal, C.; Mitch, C. H.; Knopp, K. L.; Anderson, W. H.; Cramer, J.W.; McKinzie, D. L. LY2456302 is a novel, potent, orally-bioavailable small molecule kappa-selective antagonist with activity in animal models predictive of efficacy in mood and addictive disorders. *Neuropharmacology* **2014**, *77*, 131-144.
4. Black, S. L.; Chauvignac, C.; Grundt, P.; Miller, C. N.;Wood, S.; Traynor, J. R.; Lewis, J. W.; Husbands, S. M. (2003). Guanidino N-substituted and N,N-disubstituted derivatives of the kappa-opioid antagonist GNTI. *J. Med. Chem.*, **2003**, *46*, 5505-5511.
5. Guerrero, M.; Urbano, M.; Kim, E.-K.; Gamo, A.M.; Riley, S.; Abgaryan, L.; Leaf, N.; Van Orden, L.J.; Brown, S.J.; Xie, J.Y. Design and synthesis of a novel and selective kappa opioid receptor (KOR) antagonist (BTRX-335140). *J. Med. Chem.* **2019**, *62*, 1761-1780.
6. Joshi, A. A.; Murray, T. F.; Aldrich, J. V. Structure-Activity relationships of the peptide kappa opioid receptor antagonist zyklophin. *J. Med. Chem.*, **2015**, *58*, 8783-8795.
